# Drug supply shortages and their consequences for the medical profession: a survey in Germany and Austria

**DOI:** 10.1007/s00210-026-05484-6

**Published:** 2026-06-06

**Authors:** Julia Maria Rotter, Roland Seifert

**Affiliations:** Institute of Pharmacology, Hannover Medical School, Carl-Neuberg-Straße 1, 30625 Hannover, Germany

**Keywords:** Drug supply shortage, Prescription behaviour, Survey, Time expenditure, Penicillins, Germany, Austria

## Abstract

Drug supply shortages are increasingly becoming a critical topic in the treatment of patients. Not only the reasons for the supply shortages are important but also their consequences for the medical profession. Firstly, this paper identifies which drug shortages have particularly affected medical doctors. Secondly, the additional time required for medical doctors due to supply shortages is analysed. A survey was conducted with 895 physicians from Germany and Austria using a purposive sampling method. The survey targets the supply shortages and its consequences for the medical profession of 20 commonly used drugs. All survey questions referred to the time span from November 2022 to January 2024. Penicillin V and amoxicillin emerged as the drugs most seriously affected by drug supply shortage although the severity to which physicians were affected by the individual 20 drug shortages varied regionally (Germany/Austria), depending on the place of work (practice/clinic) and the physicians’ specialty. Drug shortages reported by survey participants did not necessarily match official reporting of drug shortage. There was also a substantial increase in time required for medical doctors handling drug shortages. The most additional time burden per affected drug was recognised in haematology/oncology with 46 min, followed by psychiatry (37 min), nephrology (37 min), and general medicine (28 min). The identification of specific critical drugs, specific disciplines, and defined time-consuming factors offer starting points for improvements. Moreover, exact reporting of drug shortages by official authorities is important. Encouraging more judicious prescribing practices may help reduce perceived shortages.

**Supplementary Information:**

The online version contains supplementary material available at 10.1007/s00210-026-05484-6.

## Introduction

The topic of supply shortages of drugs has become more and more important in recent years. The question arises as to why a significant number of drugs are not available, and what underlying factors are responsible for this phenomenon (Weidenauer, 2020 p.70). Many causes contribute to these drug shortages we are facing recently. One reason is the concentration of production on a few manufacturers producing abroad. If they fail, for example due to quality issue of the raw materials, there would be a shortage of several drugs (März 2023). Another reason is just-in-time production and delivery, which does not allow flexibility for increased demand. In addition, there are discount agreements in Germany with health insurance companies that fix the price of certain medicines. Through regulation mechanisms of the free market, it is understandable that manufacturers sell to the buyers who pay the most money (März 2023). Some companies would make a conscious decision to stop producing certain drugs when patent protection expires because they are not financially worthwhile. And all these factors combined lead to the ongoing supply shortages (März 2023). The causes of supply shortages are therefore not easy to remedy overnight, and thus neither are the supply shortages themselves. It is therefore also important to develop a concept to better handle supply shortages in the long term. Numerous approaches to solutions are described already, which also include short-term measures such as the installation of early warning systems, larger warehousing, stress tests for vital products, increased transparency for authorities, and all professional circles and finally supply contracts instead of discount contracts (Weidenauer, 2020 p.100–104). But what measures make sense to support physicians who are directly affected by supply shortages? Family doctors’ practices are struggling every day with massive supply shortages for many important and widely used drugs (Deutsches Ärzteblatt 2023a). In this context, it is also interesting to know from which drug shortages they are affected the most and if there are differences depending on the work setting or country/region? How much additional time was required for physicians due to supply shortages and what caused it? In the following, an attempt is made to answer these questions by evaluating a survey of 20 selected drugs answered by physicians practising in Germany and Austria.

## Material and methods

### General information about the creation of the survey

The survey targets the supply shortages and its consequences for physicians of 20 selected drugs named amoxicillin, amoxicillin + clavulanic acid, penicillin V (phenoxymethylpenicillin), cefuroxime, cefaclor, erythromycin, cotrimoxazole, ibuprofen, paracetamol, urapidil, metoprolol, amlodipine, candesartan, tamoxifen, methotrexate, fluoxetine, lorazepam, human insulin, salbutamol, and prednisolone. The restriction to 20 drugs was chosen so that the size of the data set is sufficient to gain a good insight into the consequences of the supply shortage and at the same time the effort for the survey participant is kept to a minimum. Of the drugs, all can be found in the WHO Model List of Essential Medicines except for urapidil and cefaclor (World Health Organisation (WHO) 2023). The Union List of Critical Medicines (ULCM) also includes most of the 20 drugs except urapidil, cefaclor, ibuprofen, metoprolol, amlodipine, candesartan and fluoxetine (European Medicines Agency, Heads of Medicines Agencies, European Commission 2023).

**Fig. 1:**
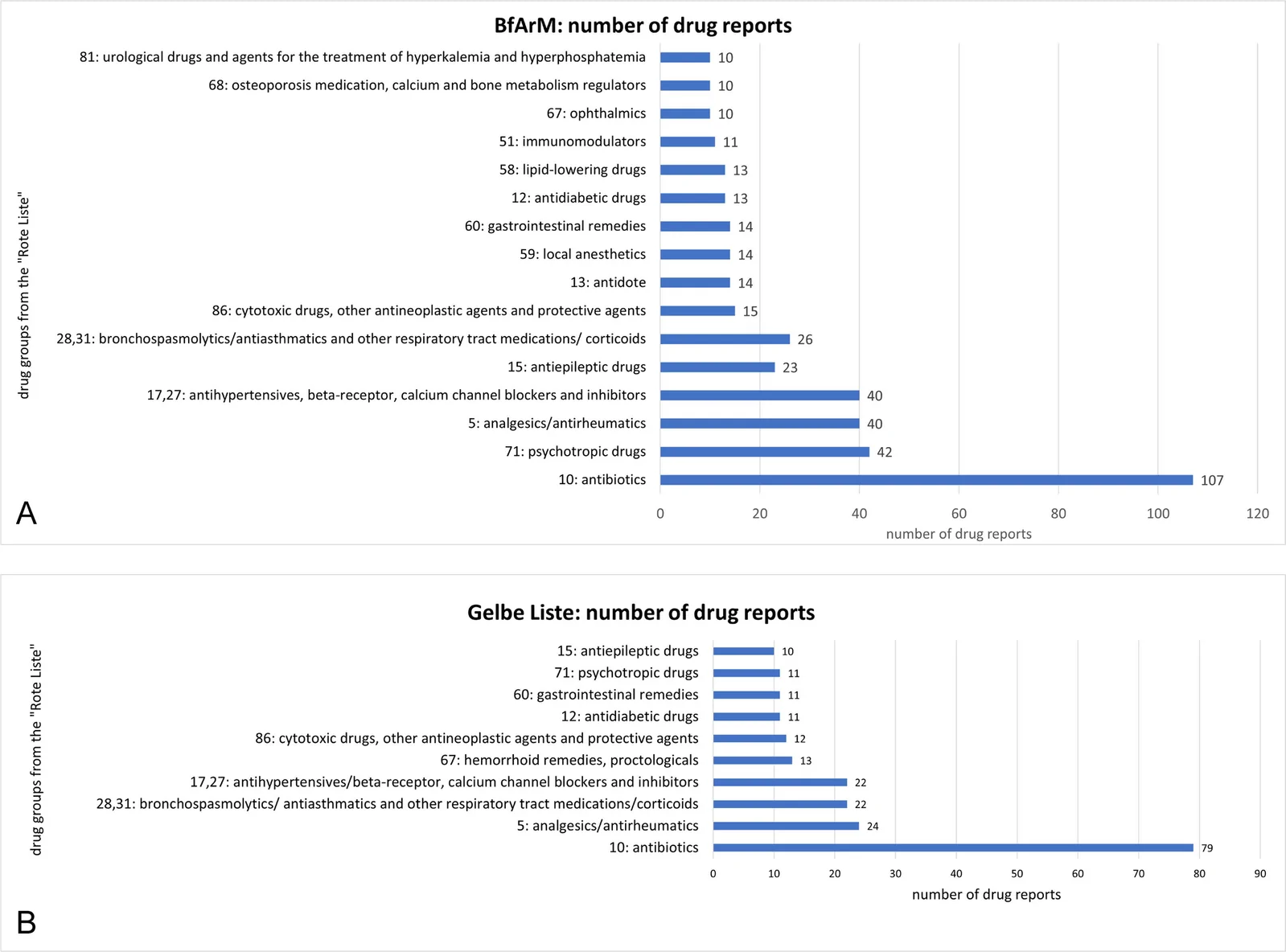
**A**, **B** Number of drug shortage reports registered by the BfArM (**A**) (status from the 7th of October 2023) and by the Gelbe Liste  2023) (**B**) (status from the 5th of November 2023) structured into the drug groups from the Rote Liste. On *y*-axis, the Rote Liste drug group number is listed. On *x*-axis, the number of drug reports classified into the Rote Liste groups are listed

Basis for the selection of the drugs was the data base of BfArM (Bundesinstitut für Arzneimittel und Medizinprodukte 2024), where pharmaceutical companies subject to the self-commitment to report supply shortages, which was decided as part of the Pharma Dialogue 2016. The self-commitment applies to all medicinal products with drugs that are on the “Liste der versorgungskritischen Wirkstoffe nach § 52b Abs. 3c AMG”(Bundesinstitut für Arzneimittel und Medizinprodukte (BfArM) 2025a, 2025b). Products with prescription drugs and a market share of 25% and more or products that are subject to the reporting obligation to hospitals (§52b paragraph 3a of the German Medicines Act) must also be reported. (Bundesinstitut für Arzneimittel und Medizinprodukte (BfArM) 2024). In addition, the “Gelbe Liste” was used for collecting information about supply shortages (Fig. 1).

For the survey, drugs were selected from the groups with the most reports in the BfArM register. This is the official register of the Federal Institute for Drugs and Medical Devices in Germany and was therefore used as a priority. The Gelbe Liste was used as a supplement. Consequently, the groups antibiotics, psychotropic drugs, analgesics, antihypertensives, antiasthmatic drugs, cytotoxic drugs, and antidiabetics were limited. Antiepileptic drugs were excluded because no reports for this group were registered in the Gelbe Liste. Drugs were selected from each of the 7 groups according to the criteria presented in Table 1.

**Table 1:** Selection criteria of the 20 drugs from the 7 groups (divided into the groups of the Rote Liste) with the most notifications in the BfArM and the Gelbe Liste with the exception of group 15

Drug groups	Chosen drug	Reason
Antibiotics/antibacterial drugs (10)	Amoxicillin	Most notifications for antibiotics in BfArM and the Gelbe Liste
	Amoxicillin + clavulanic acid	
	Penicillin V	
	Cefuroxime	Cephalosporins with the most registrations in the BfArM
	Cefaclor	
	Erythromycin	Macrolide with the most registrations in the BfArM together with azithromycin. More reports of erythromycin were hospital-relevant
	Cotrimoxazole	Representative for inhibitors of tetrahydrofolic acid synthesis (antibiotic group with the most reports apart from the groups already mentioned)
Analgesics/antirheumatics (5)	Ibuprofen	1 registration in BfArM and in Gelbe Liste—> Media presence (Deutsche Apotheker Zeitung 2023)
	Paracetamol	3 registrations in BfArM and 0 in Gelbe Liste—> Media presence (Deutsches Ärzteblatt 2022)
Antihypertensives/beta-receptor, calcium channel blockers and inhibitors Antihypertonika (17,27)	Urapidil	Most notifications for antihypertensives in BfArM
	Metoprolol	Representing group b (Seifert 2018)
	Amlodipine	Representing group c (Seifert 2018)
	Candesartan	Representing group a (Seifert 2018)
Cytotoxic drugs, other antineoplastic agents, and protective agents (86)	Tamoxifen	Most notifications in BfArM for cytostatic drugs
	Methotrexate	In comparison to the other drug with the most notifications named topotecan it is not only used for not only approved for the treatment of cancers. In addition, low-dose methotrexate is considered the gold standard in the treatment of rheumatoid arthritis. (Freissmuth et al. 2024, p. 835)
Psychotropic drugs (71)	Fluoxetine	According to the Professional Association for Child and Adolescent Psychiatry, Psychosomatics and Psychotherapy in Germany (BKJPP), no other drug is approved for children and adolescents for the treatment of severe forms of depressive disorders. (Deutsches Ärzteblatt 2023b)
	Lorazepam	Reported in BfArM and Gelbe Liste unlike alprazolam
Antidiabetic drugs (12)	Human insulin	Most reports for antidiabetic drugs
Bronchospasmolytics/antiasthmatics and other respiratory tract medications/corticoids (internals) (28,31)	Salbutamol	The most frequently mentioned drugs from the Rote Liste Group 28; 31
	Prednisolone	

#### Structure of the questionnaire

All survey questions referred to the time span from November 2022 to January 2024. The first block of questions are general questions, including federal state of the survey participants’ workplace, their specialisation, and the setting (doctor’s office, clinic, company, else) they work in. In the second block of questions, participants are asked how affected they had been by the supply shortages of the 20 drugs. In the third block of questions, the participants were asked further questions on the drugs they were affected the most in their practical field. Further questions included which dosage form was affected the most and which alternative they had chosen for most of the patient cases. The fourth and last block of questions covers the increased time consumption that follows supply shortages.

This paper focuses on the question blocks 1, 2, and 4, as considering all the results would go beyond the scope of this paper. The results of question block 3 have been recently published in (Rotter and Seifert 2026).

Three personal questions are asked in the survey. But just with the federal state of the work place, the specialty and the work setting it is not possible to draw conclusions about individual persons from the data set. Therefore, the survey is anonymous. In addition, it was possible to select “not specified” in the personal questions and still take the survey.

Data protection: Data protection within the meaning of the DSGVO (Datenschutz-Grundverordnung/General Data Protection Regulation) does not apply at this point because the survey is anonymous.

Ethics approval: According to the Ethics Committee of the Hannover Medical School, anonymous surveys are not subject to consultation, as they do not collect any personal data. Therefore, no ethics application was necessary for the survey.

Table S1 shows the survey distributors and Table S2 shows the questionnaire translated into English. The questions were asked in German language.

##### General information about the realisation of the survey

The survey was carried out with the programme SoSci survey with a license from the “Medizinische Hochschule Hannover”. The survey was pretested with a small number of university colleagues and physicians including the verification of selection ranges and time intervals.

The survey was released on the 7th of January 2024 and closed on the 4th of August 2024. Seventy-five contacts in Germany and 26 contacts in Austria were asked to distribute the survey including different types of medical associations. Eighteen German contacts and 9 Austrian contacts finally answered and helped to distribute the survey.

There were no inclusion criteria whether respondents needed to have prescribed the drugs or experienced shortages.

In total, 895 survey participants were analysed. Therefore, the number of survey participants is significant because it is higher than the minimal sample size of 384. The minimal sample size was measured with the following equation: $n=\frac{{z}^{2}\times p (1-p)}{{E}^{2}}$

*n* = sample size.

*z* = 1.96 for a 95% confidence level.

*p* = share of the characteristic in the population: In this case, this proportion is not known and is therefore assumed to be the maximum value of 0.5

*E* = margin of error: Has been set to a value of 0.05, so that the result can differ by a maximum of 5% from the actual result of the population.

##### General information about the analysis of the survey

The analysis of the survey response data has been performed in Python within a Jupyter Notebook utilising pandas, numpy, and matplotlib. Incomplete answers to question blocks were excluded. Specific exclusions of small number groups are described in the respective figures. No further inferential statistics has been used.

To collapse the histogram of the rating how strongly being affected by the shortage into one number, a measure for being affected by supply shortages has been defined: Percentage of participant being “strongly” or “very strongly affected” by the respective shortages, i.e. the top 2 selection categories.

In Figs. 5, 6, 7, 8, 9, 10, 11, 12, 13, and 14, the confidence interval of 95% is described as a vertical line in each column.

The confidence interval of 95% is calculated based on the margin of error with the following equation: ${\mathrm{M}\mathrm{O}\mathrm{E}}_{95}\approx 1.96 \sqrt{\frac{{\sigma }^{2}}{n}}$

The following medical hypothesis was put forward:

Null hypothesis H0: Drug supply shortages do not lead to additional time expenditure for medical doctors.

Alternative hypothesis H1: Drug supply shortages lead to additional time expenditure for medical doctors.

## Results

### Participant demographics of the survey

Six hundred sixty-five survey participants stated to be practising in Germany and 190 survey participants stated to be practising in Austria.

The following pie charts show the distribution of survey participants by federal state in percentage.

**Fig. 2:**
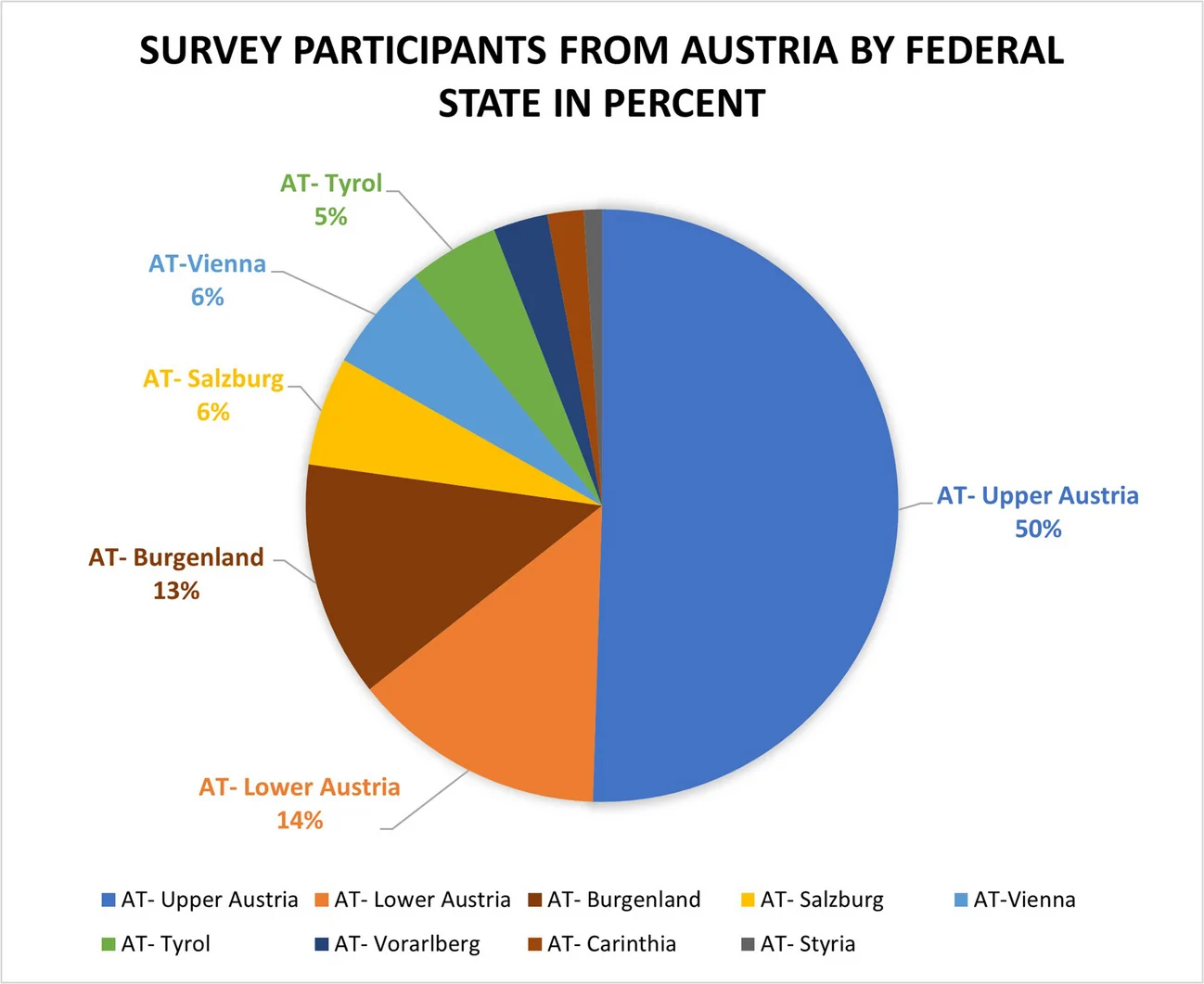
Distribution of survey participants from Austria by federal state

**Fig. 3:**
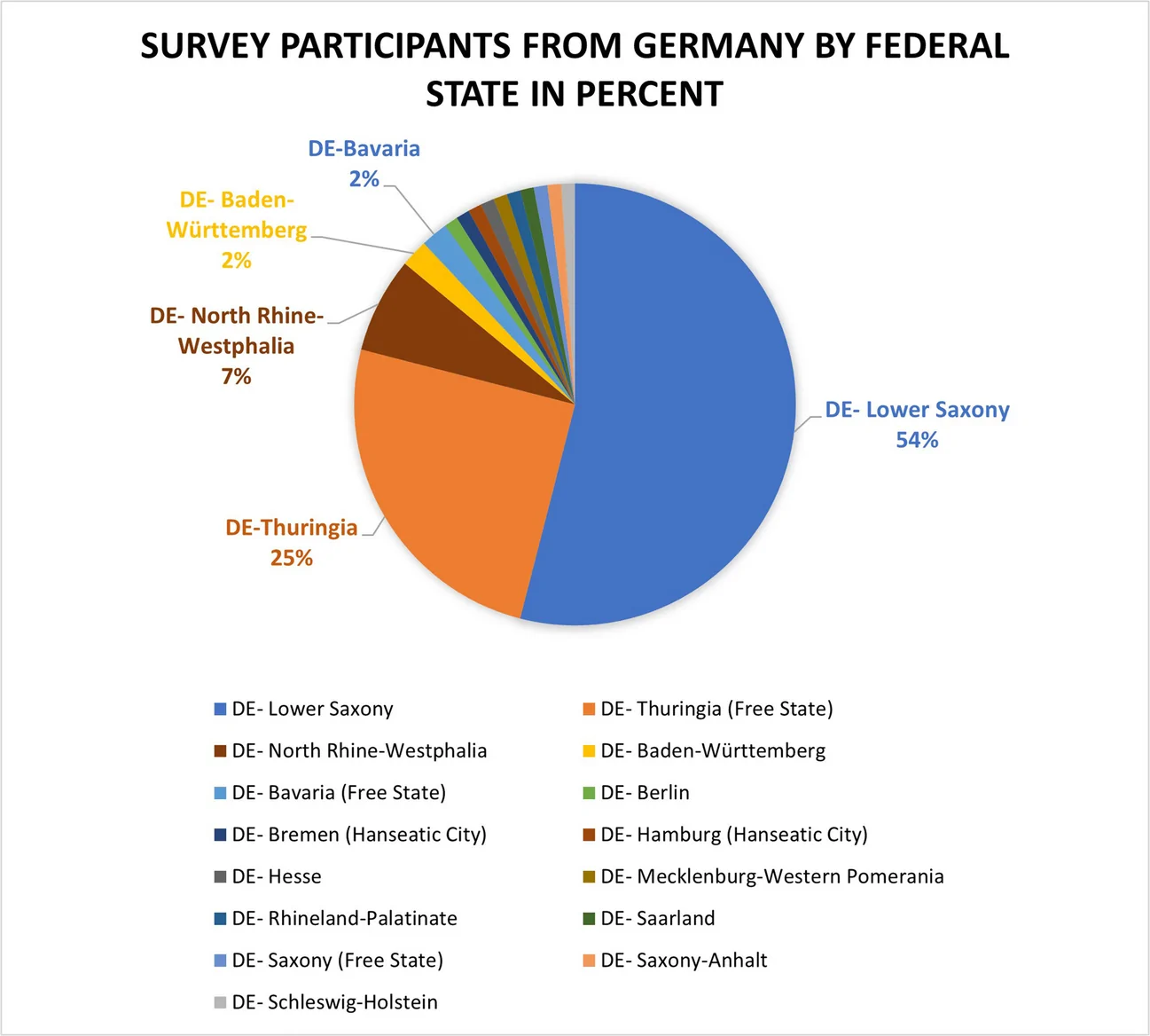
Distribution of survey participants from Germany by federal state

Half of the survey participants from Austria practise in Upper Austria. Physicians from the other federal states also participated, but to a much smaller extent (Fig. 2). More than half (54%) of the survey participants from Germany practise in Lower Saxony and 25% of the survey participants practise in Thuringia. As with the Austrian participants, however, every federal state is represented among the German participants, but some only to a very small extent (Fig. 3).

**Fig. 4:**
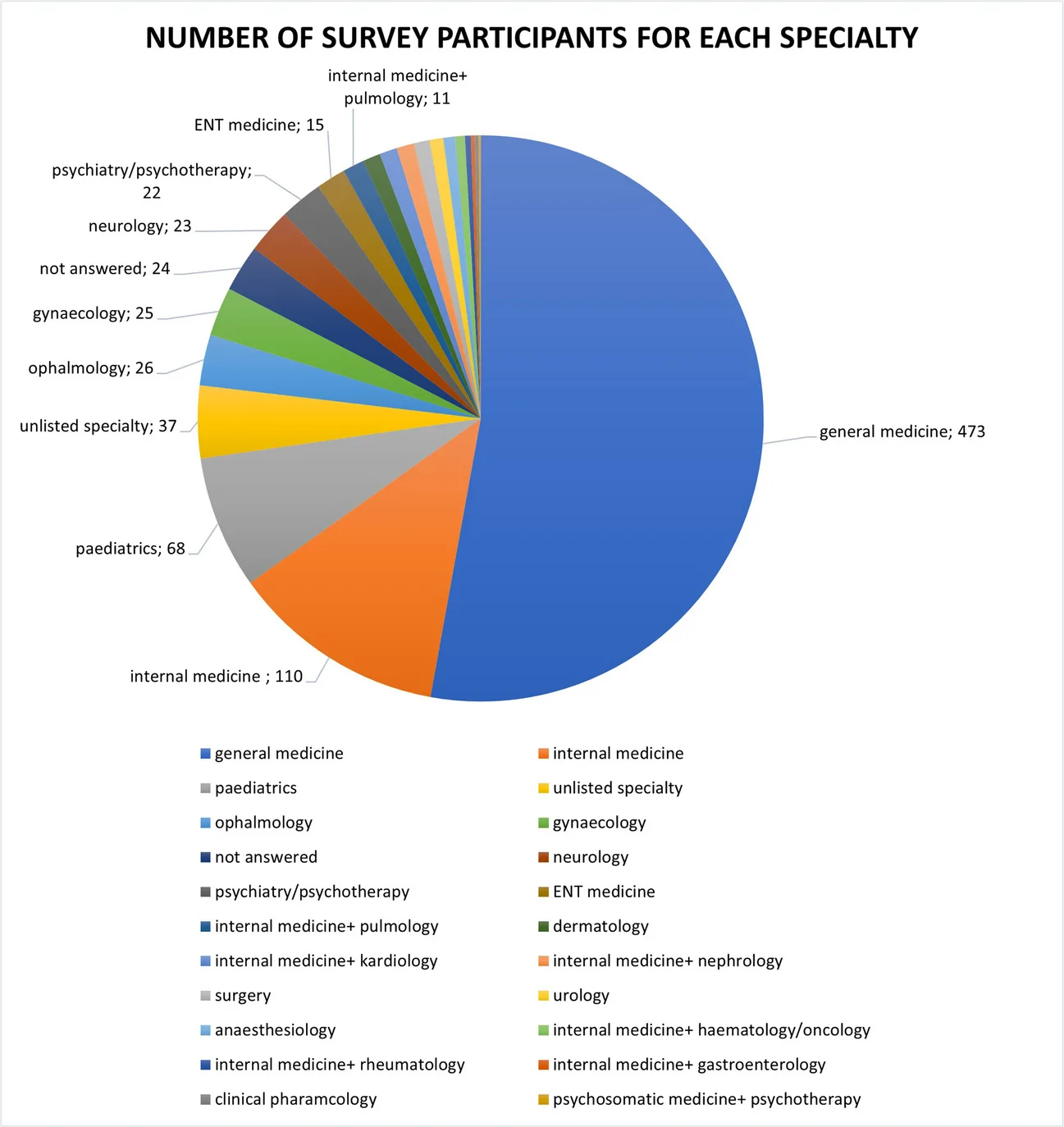
The number of survey participants for each specialty

More than half of the participants are general practitioners. Internal physicians and paediatricians form the second and third largest groups (Fig. 4).

### How physicians indicated being affected by the supply shortages of the 20 drugs

#### Overview the 4 drugs with the highest ratings

**Fig. 5:**
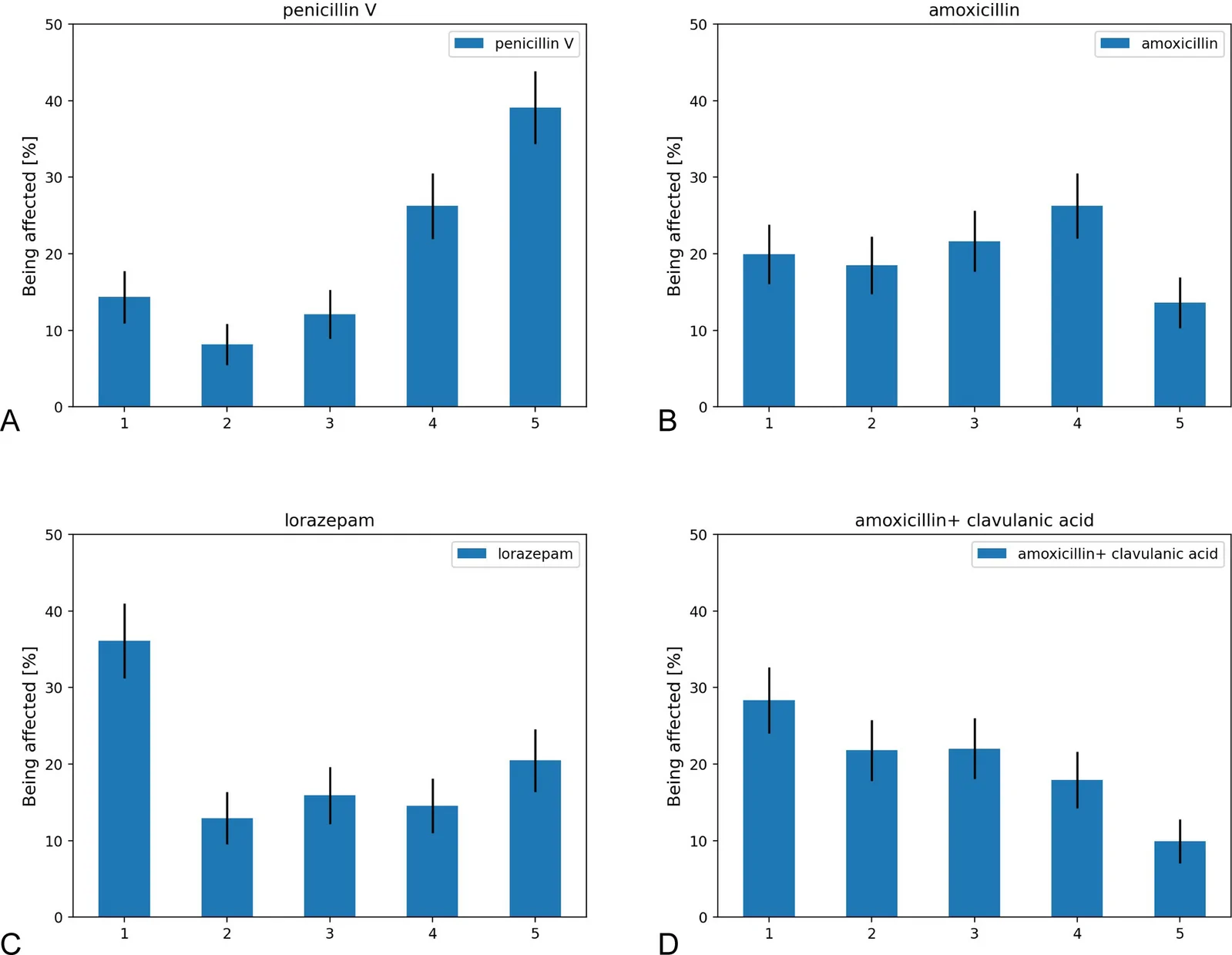
**A**–**D** Distribution of the impact of penicillin V (**A**), amoxicillin (**B**), lorazepam (**C**), and amoxicillin + clavulanic acid (**D**) supply shortages on general practitioners (largest group of survey participants) Assessment 1–5 on *x*-axis: 1 = not affected by the supply shortage of the drug, 2 = slightly affected, 3 = moderately affected, 4 = strongly affected, 5 = very strongly affected

The evaluation of the drugs penicillin V, amoxicillin, lorazepam, and amoxicillin + clavulanic acid is presented separately here, as these drugs were rated the highest of all 20 selected drugs in terms of being affected by the supply shortages. Fourteen percent (95% CI: 10.96%–17.82%) of the survey participants were not affected by the supply shortage of penicillin V, whereas 86% of the physicians were affected (Fig. 5A). Thirty-nine percent (95% CI: 34.43%–43.97%) of the participants rated being affected by the supply shortage of penicillin V even as very strongly (‘5’) (Fig. 5A). This is one big difference in comparison to the other 3 drugs. For amoxicillin, amoxicillin + clavulanic acid, and lorazepam, the percentage of participants that stated being very strongly affected was much lower, with the highest percentage of 21% (95% CI: 16.42%–24.66%) for lorazepam (Fig. 5C).

The supply shortage of amoxicillin affected not only Germany or Austria but also other European countries. In France in particular, the situation was critical due to the shortages of amoxicillin, also in combination with clavulanic acid. In Spain, in November 2022, the stock of amoxicillin could not even cover 50% of the demand. In the USA, there were also problems with the availability of amoxicillin and clavulanic acid, as well as in Romania, Malaysia, Australia, and Canada (Cohen et al. 2023). Therefore, it can be concluded that the problem of supply shortages is a global problem, at least with regard to certain drugs.

#### Similarities and differences depending on the medical specialty

**Fig. 6:**
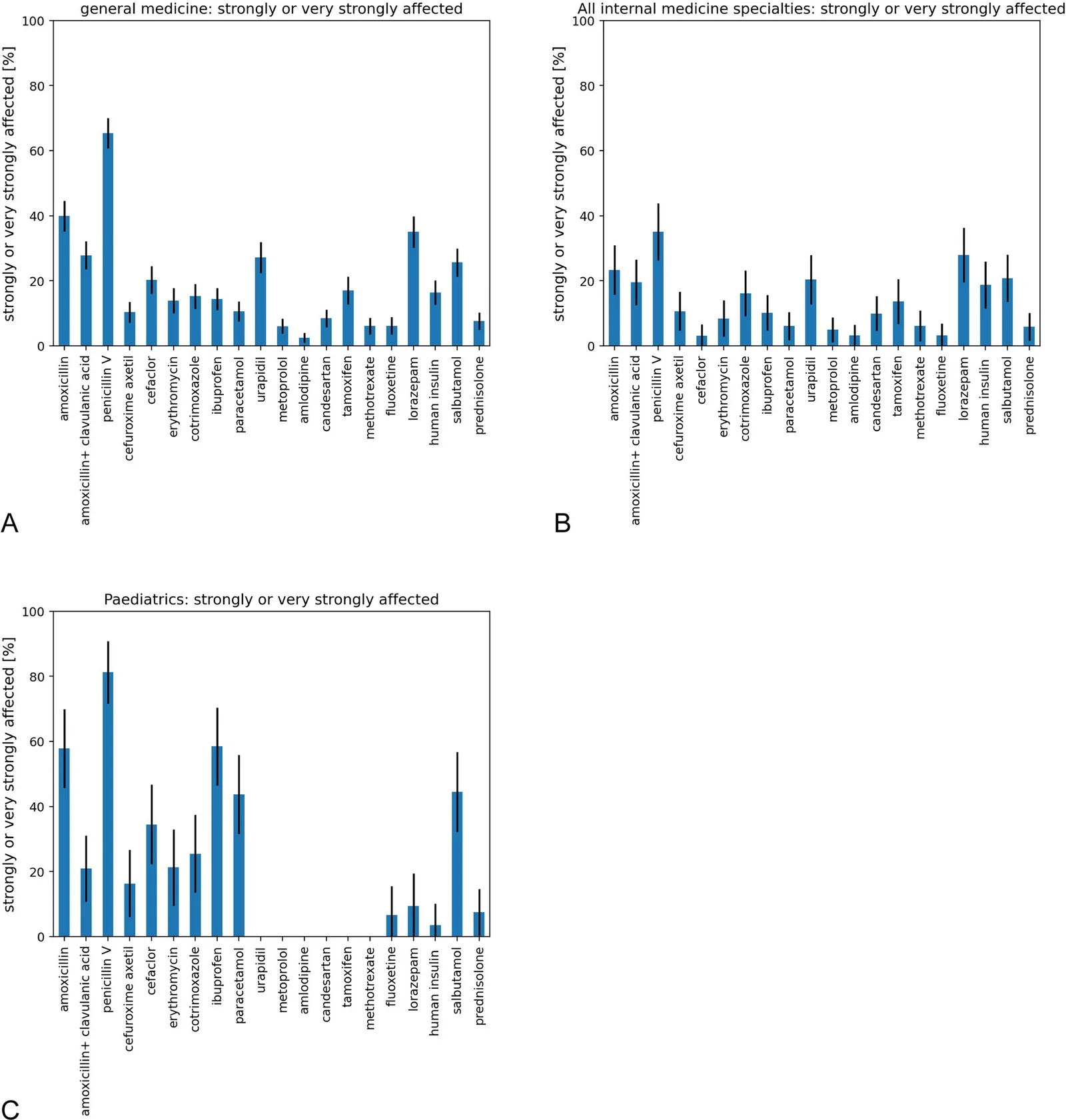
**A**–**C** Percentage of general practitioners (**A**), internal medicine physicians (**B**), and paediatricians (**C**) who were severely or very severely affected by the shortage of the individual drug

Figure 6A–C shows the percentage of survey participants who stated to be strongly or very strongly affected by the supply shortage for each of the chosen 20 drugs, per specialisation of the medical doctors.

For general practitioners (Fig. 6A), the drugs with the highest ratings are penicillin V, amoxicillin, and in third place lorazepam. General practitioners stated to be particularly affected by the supply shortage of penicillin V (65%) (95% CI: 60.61%–69.91%), amoxicillin (40%) (95% CI: 35.26%–44.74%), and amoxicillin + clavulanic acid (28%) (95% CI: 23.58%–32.24%), whereas only 20% (95% CI: 15.77%–24.35%) stated to be strongly or very strongly affected by the supply shortage of cefaclor. Less than 20% of the general practitioners stated to be strongly or very strongly affected by the supply shortage of cefuroxime, erythromycin, and cotrimoxazole. The percentage of the general practitioner stating to be strongly or very strongly affected by the supply shortage of the analgesic substances ibuprofen and paracetamol is also very small with under 20% for both substances.

Looking at the antihypertensive drugs (urapidil, metoprolol, amlodipine, candesartan), the general practitioners were affected to varying degrees by the supply shortage of these drugs.

Twenty-seven percent (95% CI: 22.47%–32.07%) of the general practitioners were strongly or very strongly affected by the supply shortage of urapidil. Being strongly or very strongly affected by the supply shortage of the other three drugs named metoprolol, amlodipine, and candesartan is stated to be less than 10%.

The general practitioners stated to be less affected by the supply shortage of salbutamol with 25% (95% CI: 21.18%–29.7%) than for urapidil with 27%. Tamoxifen and insulin human have also a similar rating with 17% (95% CI: 12.58%–20.98%) for tamoxifen and 16% (95% CI: 12.7%–20.1%) for insulin human. For methotrexate, fluoxetine, and prednisolone, only less than 10% of the general practitioner stated to be strongly or very strongly affected by the supply shortage of these drugs.

**Fig. 7:**
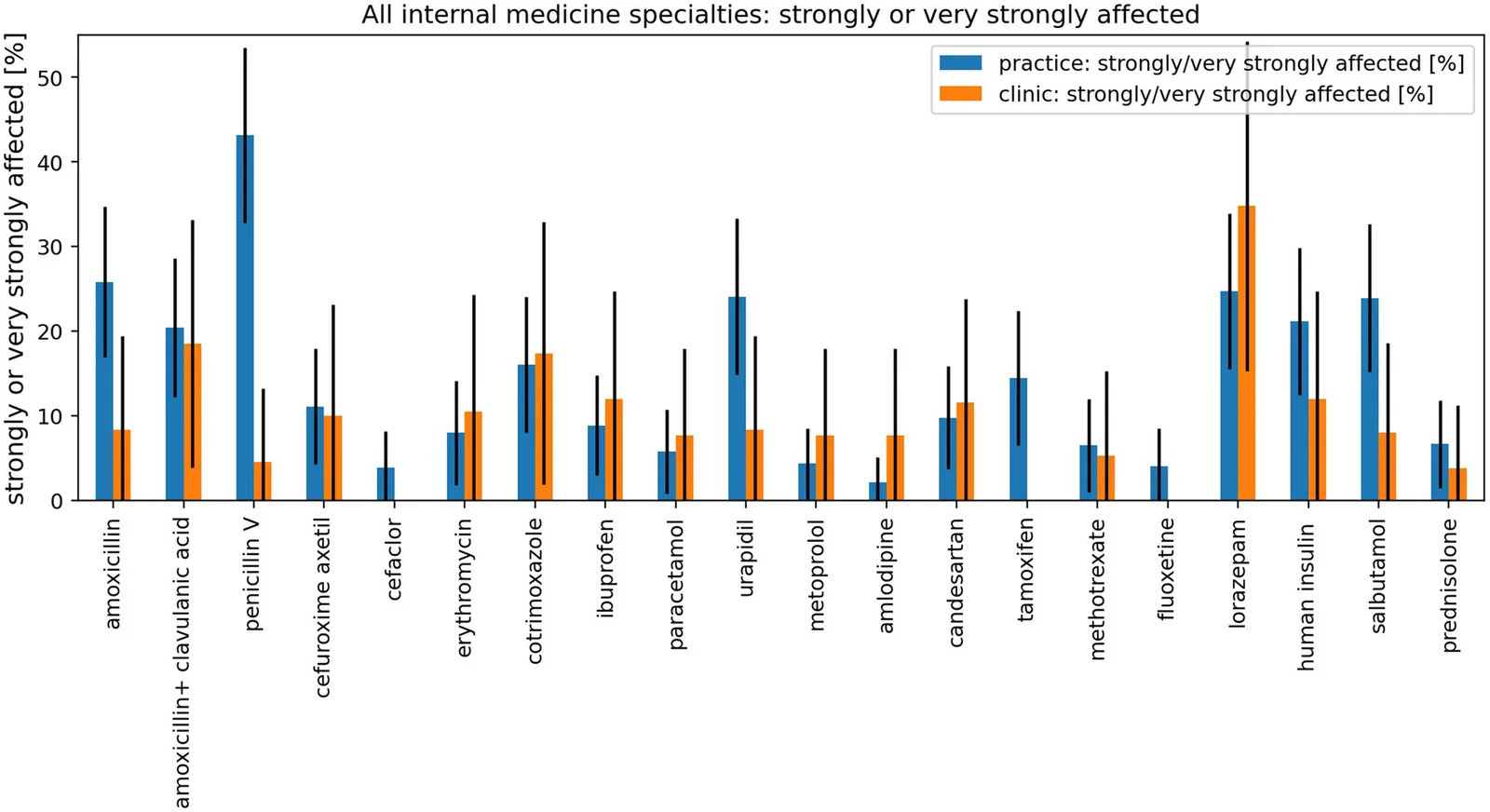
Percentage of internal medicine physicians who were strongly or very strongly affected by the shortage of the individual drug. Comparison of internal medicine physicians working in a medical practice (112 survey participants) vs. internal medicine physicians working in a clinic (35 survey participants)

Comparing the general practitioners (Fig. 6A) with the internal medicine (Fig. 6B), the following similarities and differences can be seen: with only one percentage point difference, the general practitioner and the medical doctors with the specialty internal medicine rated to be equally affected by the supply shortage of the drugs cefuroxime, cotrimoxazole, metoprolol, amlodipine, candesartan, methotrexate, and prednisolone. For ibuprofen, paracetamol, tamoxifen, salbutamol, and fluoxetine, the general practitioners were slightly more affected than the medical doctors with the specialty internal medicine. With a variation of 6–8%, the general practitioners are more affected by the supply shortage of erythromycin, urapidil, lorazepam, and amoxicillin + clavulanic acid. The biggest difference between both specialties was observed for amoxicillin, cefaclor, and penicillin V. Forty percent of the general practitioners stated to be strongly or very strongly affected by the supply shortage of amoxicillin whereas only 23% (95% CI: 15.76%–30.9%) of the physicians working in the internal medicine specialty stated to be strongly or very strongly affected by the supply shortage of amoxicillin. One possible explanation that the general practitioners where more affected by the shortage of amoxicillin could be that amoxicillin is prescribed less frequently in hospitals than in outpatient settings (Federal Office of Public Health FOPH 2024). General practitioners usually work in an outpatient setting. However, the internists are also partly active in the hospital and are therefore less affected than the general practitioners. The difference between outpatient and inpatient settings is also illustrated in Fig. 7. Looking at the drug cefaclor, it can be seen a similar tendency. The largest difference can be seen for penicillin V. Sixty-five percent of the general practitioners stated to be strongly or very strongly affected by the supply shortage of penicillin V whereas only 35% (95% CI: 26.33%–43.85%) of the internal medical doctors stated to be strongly or very strongly affected by the supply shortage of penicillin V.

Comparing the general practitioners to the paediatricians (Fig. 6C), the following similarities and differences can be seen: with only one percentage point variation the general practitioner and paediatricians rated to be equally affected by the supply shortage of fluoxetine and prednisolone. Looking at the ratings of lorazepam and insulin human, the general practitioners stated to be more affected by the supply shortages of these drugs in comparison with the paediatricians. The paediatricians were more affected by the supply shortages of amoxicillin, penicillin V, ibuprofen, paracetamol, and salbutamol. For ibuprofen and paracetamol, the ratings show a particularly big difference. 59% (95% CI: 46.48%–70.44%) of the paediatricians rated to be strongly or very strongly affected by the supply shortage of ibuprofen whereas only 14% (95% CI: 10.8%–17.56%) of the general practitioners rated to be strongly or very strongly affected by the supply shortage of ibuprofen. The same tendency can be seen for paracetamol with a value of 44% of the paediatricians and a value of 11% (95% CI: 7.59%–13.67%) for the general practitioners.

In general, the fields of work of internists and general practitioners differ, which lead to different usage of drugs and therefore the physicians are being affected by the drug shortages differently regarding their specialty.

#### Similarities and differences depending on the work setting of the medical doctors

There are differences between how the internal physicians were affected by the supply shortage of the 20 drugs between the medical doctors working in a doctor’s office and physicians working in a clinical setting (Fig. 7). The number of survey participants working in clinics was very small. Therefore, it is not possible to make a statement about all 20 drugs. The only drug with a statistically significant difference between the two groups is penicillin V. Over 40% of physicians working in a doctor’s office stated to be strongly or very strongly affected by the supply shortage of penicillin V whereas only 5% of medical doctors working in clinics stated to be strongly or very strongly affected by the supply shortage of penicillin V. For amoxicillin and salbutamol, survey participants working in a doctor’s office stated to be more affected by the supply shortage of amoxicillin and salbutamol than the survey participants working in clinics.

#### Regional similarities and differences

**Fig. 8:**
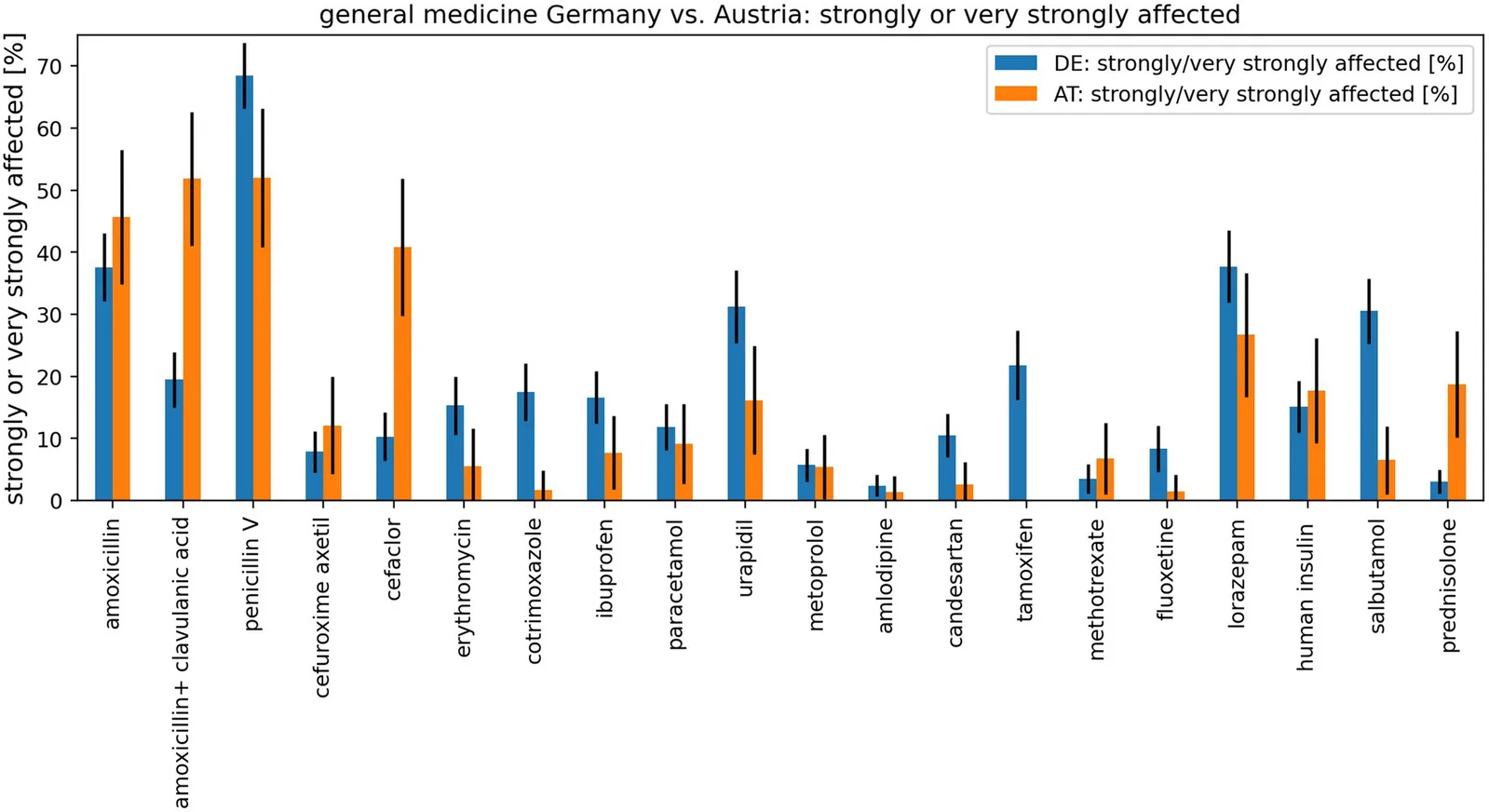
Percentage of general medicine physicians practising in Germany vs. Austria who were strongly or very strongly affected by the shortage of the individual drugs

We differentiated between general practitioners working in Germany and in Austria to picture regional differences (Fig. 8). General practitioners working in Germany stated to be more affected by the supply shortage of penicillin V, erythromycin, urapidil, and salbutamol in comparison to the general practitioners working in Austria. General practitioners working in Austria stated to be more affected by the supply shortage of amoxicillin + clavulanic acid, cefaclor, and prednisolone in comparison to the general practitioners working in Germany.

**Fig. 9:**
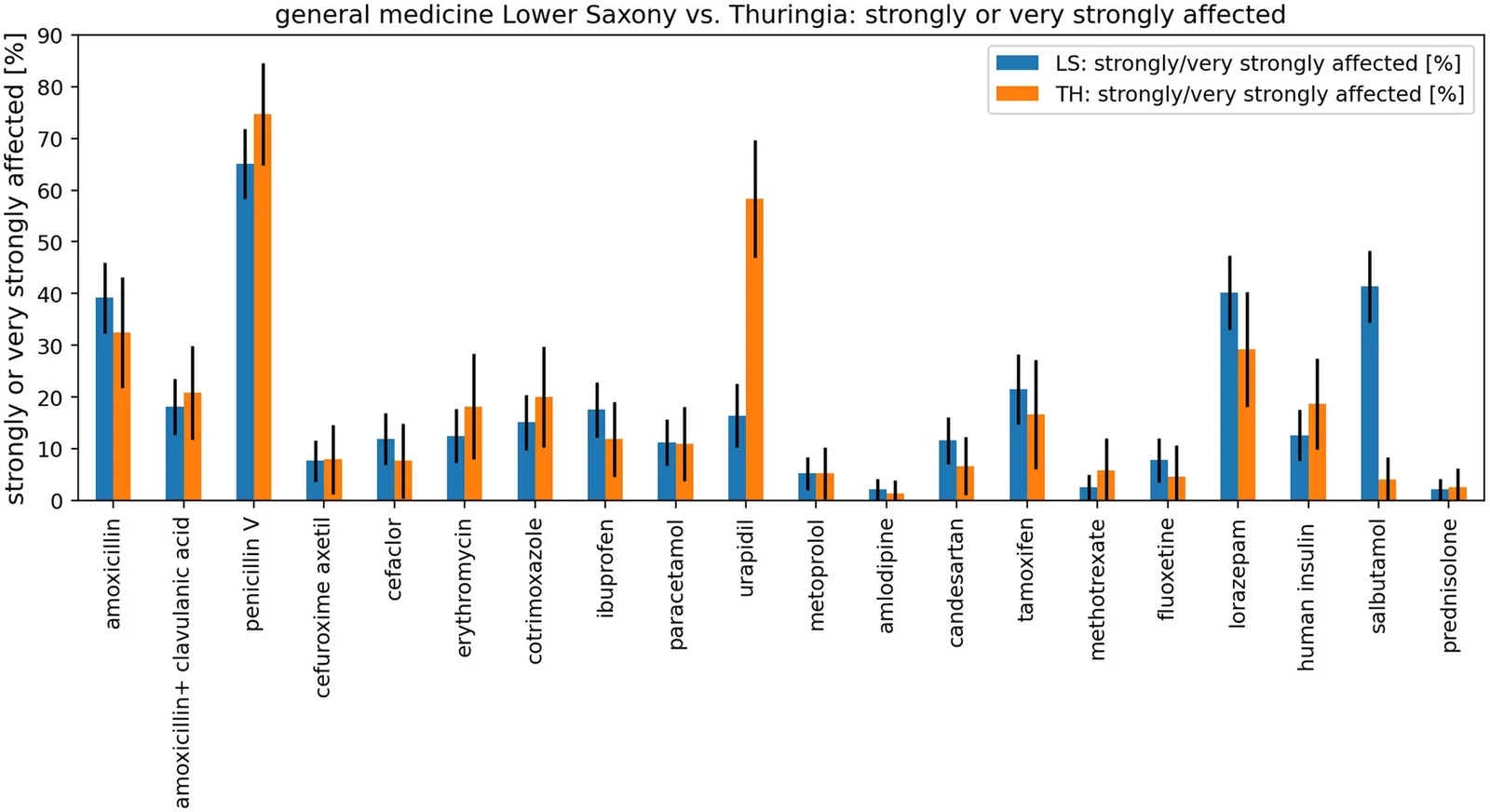
Percentage of general medicine physicians practising in Lower Saxony vs. Thuringia who were strongly or very strongly affected by the shortage of the individual drugs

We differentiated between general practitioners working in the German federal state Lower Saxony and such working in the German federal state Thuringia (Fig. 9). General practitioners working in Lower Saxony stated to be more affected by the supply shortage of salbutamol in comparison to the general practitioners working in Thuringia. General practitioners working in Thuringia stated to be more affected by the supply shortage of urapidil in comparison to the general practitioners working in Lower Saxony.

The extent to which the drug shortages are affected varies depending on the drug. It is not evident that in one federal state/state, there was a significantly greater impact on all medicinal substances than in the other federal state/state. This is the best way to assume that the differences in the percentage of physicians being affected not only between Lower Saxony and Thuringia but also between Germany and Austria are due to differences in the demand for these drugs.

##### Increased time expenditure for medical doctors due to supply shortages of the 20 drugs

In a survey in 2013 by 1070 members of the Canadian Pharmacists Association, the Canadian Medical Association, and the Canadian Society of Hospital Pharmacists “physicians and pharmacists [already] reported that the shortages were leading to inefficiencies in the workplace and taking their time and energies away from patient care. Sixty-seven percent of physicians reported an increase in time spent on research and consultation to source alternative medication—and 76% of community and hospital pharmacists indicated that drug shortages have a significant impact on their workload” (Lynas 2013, p. p.67). In the following, the additional time expenditure for in Germany and in Austria practicing medical doctors from the survey is analysed.

#### Additional time expenditure for each specialty

**Fig. 10:**
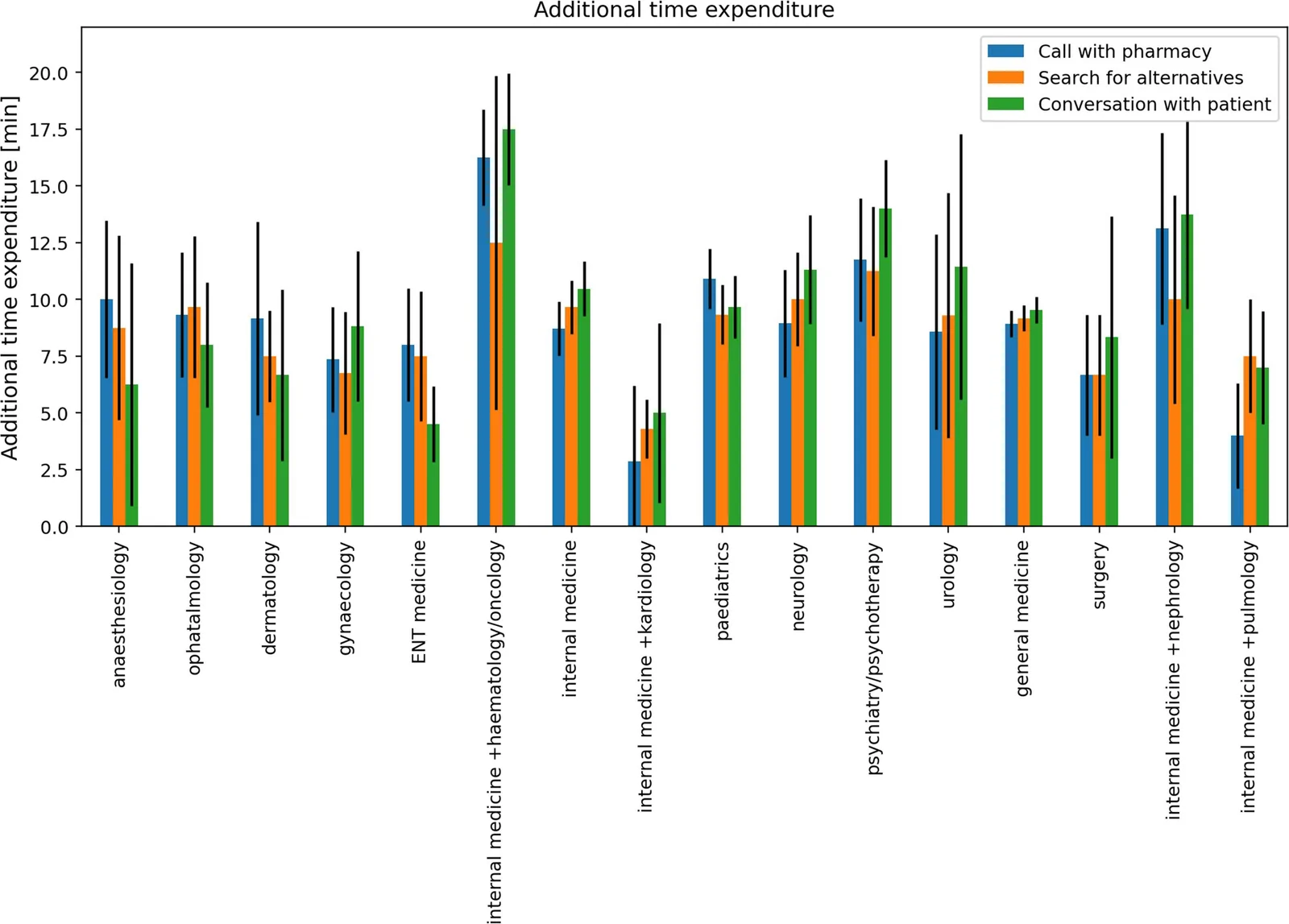
Additional time expenditure in minutes for each specialty per affected drug. Specialties with zero or one answer were not presented

Figure 10 shows the additional time for each specialty in minutes per affected drug. Three sources of additional time expenditure were queried: phone calls to the pharmacy, searching for alternatives, and speaking with the affected patient. Within the specialty, the physicians rated similar amounts of additional time for the three reasons, on average. But the additional time that was needed varies between the specialties.

#### Additional time expenditure for internal medicine practitioners in comparison to general practitioners

**Fig. 11:**
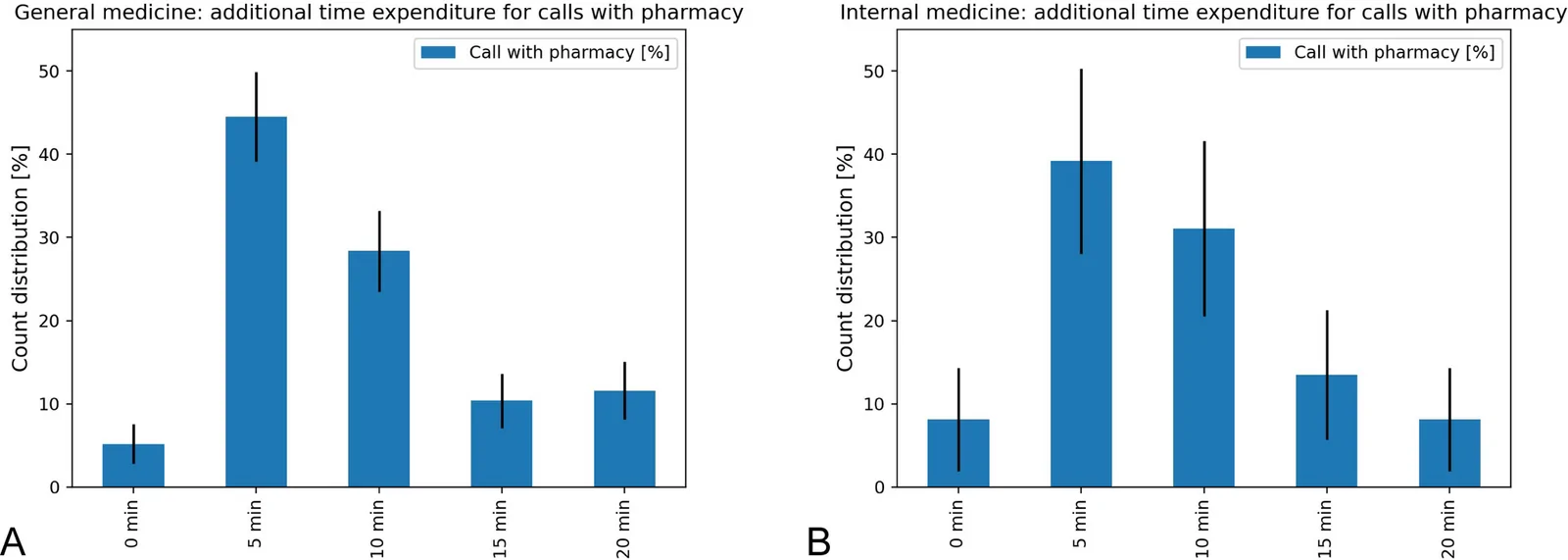
**A**, **B** Distribution of additional time expenditure for calls with pharmacy for general medicine practitioners (**A**) and for internal medicine practitioners (**B**)

**Fig. 12:**
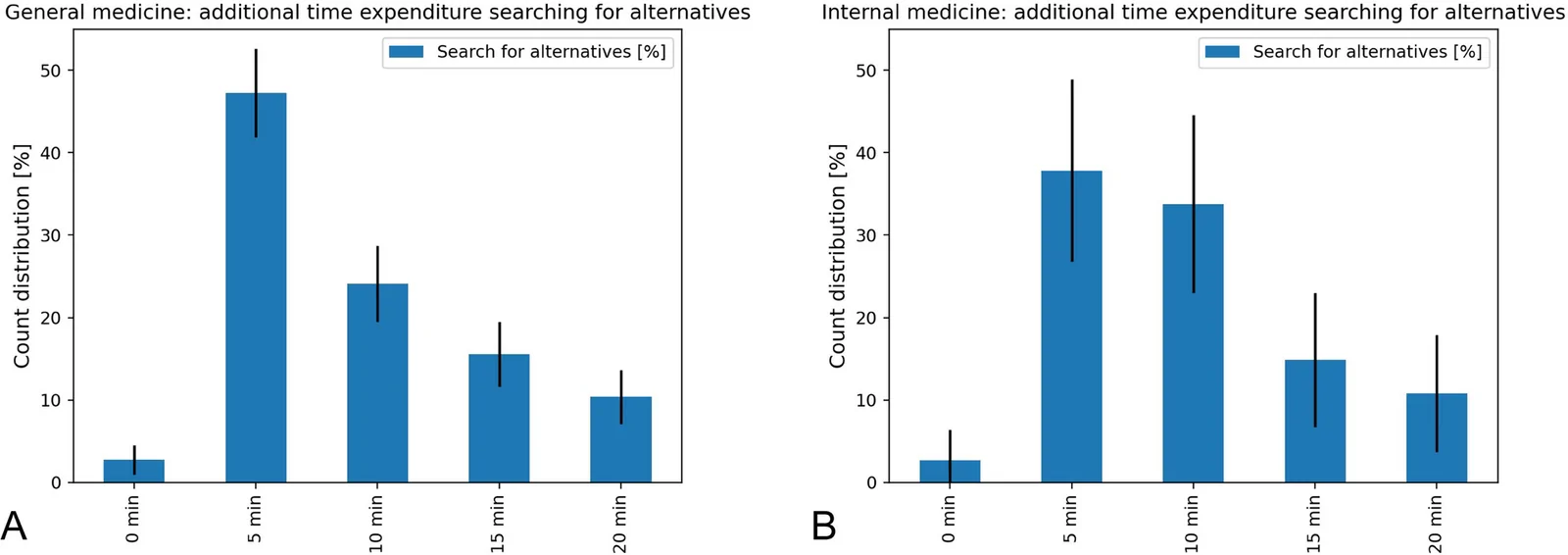
**A**, **B** Distribution of additional time expenditure for searching for alternatives for general medicine practitioners (**A**) and for internal medicine practitioners (**B**)

**Fig. 13:**
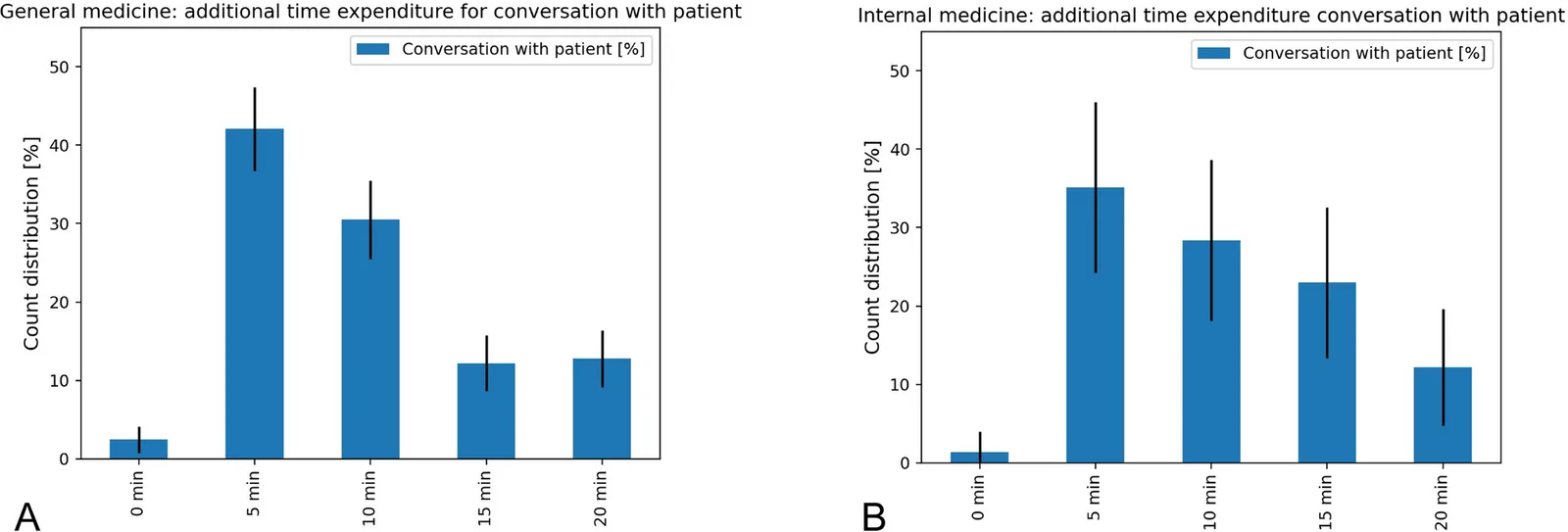
**A**, **B** Distribution of additional time expenditure for conversation with the patient for general medicine practitioners (**A**) and for internal medicine practitioners (**B**)

Figures 11, 12, and 13 show the additional time expenditure separated for each source for additional time expenditure stated by the general practitioners in comparison to the internal medical doctors.

Figure 11A, B shows the additional time expenditure per affected drug for phone calls with the pharmacy. In both specialties, the largest percentage of the physicians needed 5 min additional time per drug. Seventy-three percent of the general practitioners needed 5 to 10 min of additional time and nearly the same percentage (71%) of physicians working in internal medicine. Fifteen to 20 min additional time was needed by 22% of general practitioners and 21% of internal physicians.

Only a few percent of physicians needed no additional time for searching for alternatives (Fig. 12A, B). In both specialties, the largest percentage of the physicians needed 5 min additional time. Seventy-one percent of the general practitioners and also the same percentage of medical doctors working in internal medicine needed 5 to 10 min of additional time. Fifteen to 20 min additional time was needed by 26% of general practitioners and 25% of physicians working in internal medicine.

Only a few percent of physicians needed no additional time for speaking with the affected patient (Fig. 13A, B). In both specialties, the largest percentage of the physicians needed 5 min additional time. Seventy-two percent of the general practitioners needed 5 to 10 min of additional time but only 65% of physicians working in internal medicine. Fifteen to 20 min additional time was needed by 25% of general practitioners and by 34% of physicians working in internal medicine. For speaking with the affected patient, it is possible that doctors working in internal medicine needed more additional time than general practitioners.

#### Cumulative additional time expenditure

**Fig. 14:**
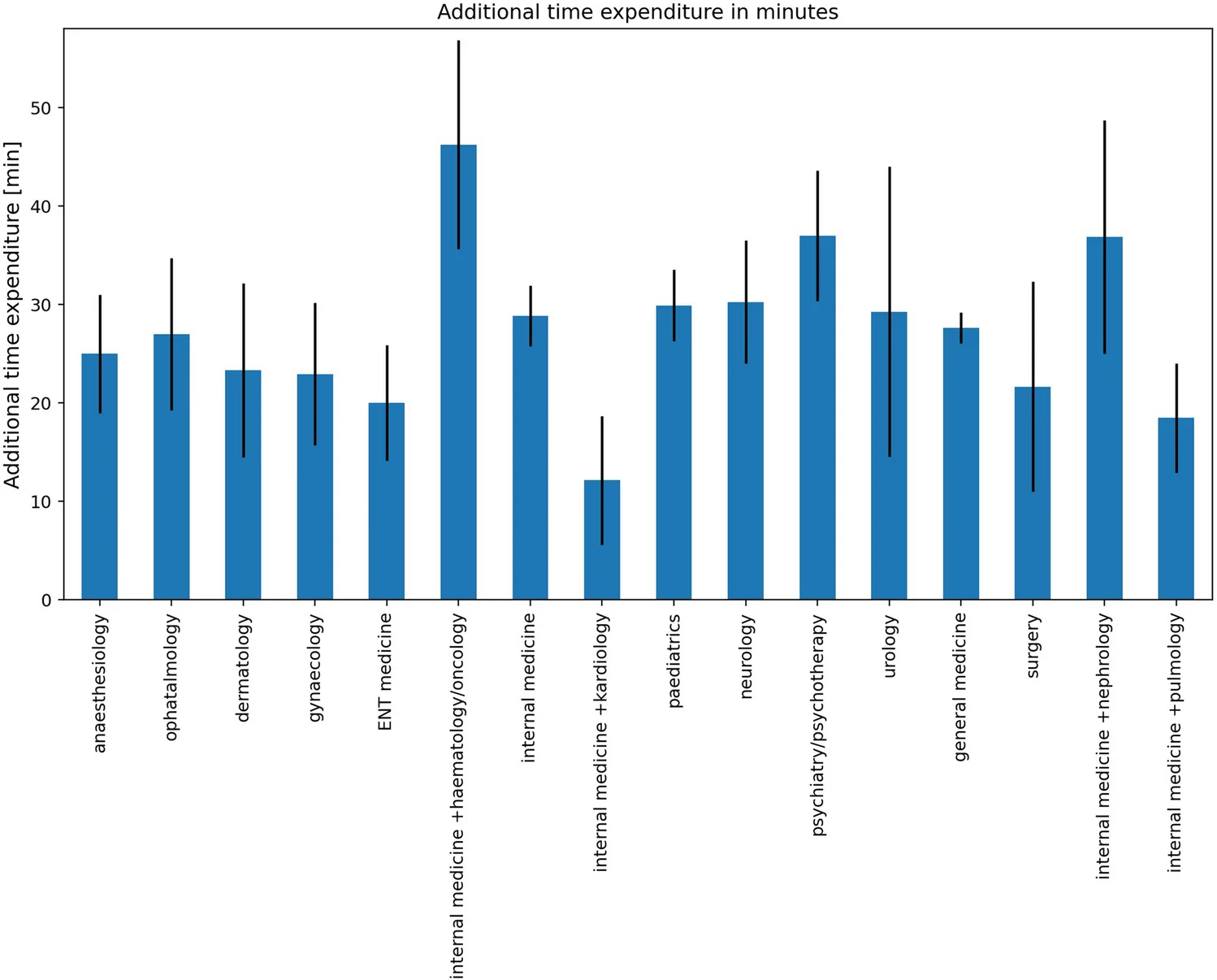
Cumulative additional time in minutes per affected drug for each specialty. Specialties with zero or one answer were not presented

Figure 14 shows the cumulative additional time for each specialty in minutes per affected drug. The three specialties with the largest amount of cumulative additional time are haematology/oncology with 46 min additional time per drug, and psychiatry, and nephrology with both 37 min of additional time per drug. From most of the specialties the cumulative additional time was rated by over 20 min per affected drug. General practitioners needed a cumulative time of 28 min per affected drug.

Based on this data, the null hypothesis can be rejected in favour of the alternative hypothesis.

#### Additional sources for additional time expenditure

Table 2 shows additional sources for time expenditure which survey participants wrote in a free text field. The two most commonly mentioned sources for additional time expenditure are the prescriptions and communication. The reason why prescriptions needed more time is because prescriptions needed to be changed if the originally prescribed drug was not available in pharmacy. Physicians had difficulties in cancelling the digitally ordered original prescription. The system with the digital prescriptions was introduced in January 2024 that means it was/is a new system (Bundesministerium für Gesundheit 2024). Additionally, some patients wanted to have a prescription in advance for drug storage. Another source for additional time expenditure is the communication with affected patients, with patients just worrying, with relatives, with nursing homes and retirement homes, with other staff members and with other physicians. Although the other sources for additional time expenditure in Table 2 were mentioned by a smaller number of physicians, they can also be helpful for finding solutions for saving time.

**Table 2:** Additional sources for additional time expenditure

*Additional sources for additional time expenditure*	*Number of participants*
Prescriptions: Change prescriptions to transfer the new prescription trouble with the electronic prescription changing PZN numbers for same pharmaceuticals wish of patients to have a prescription to stock up	50
Additional communication with: Affected patients or not affected but worried patients, dependants, nursing homes, retirement homes, the team in the medical practice, colleagues (from another specialty)	37
Searching for a pharmacy with inventory: And if necessary, generating a daily updated list of available drugs	17
Changing the medication plan/ Creating a new medication plan	14
Research Searching for availability and recommendations (minimising the risk of recourse) for drugs	11
Problems with the pharmacy: Wrong recommendations sending patients away aut idem discussions no accessibility	6
Additional documentation: Justification for prescribing the alternative drug, justification for the prescribed pack size	6
Having to make multiple appointments with the patients: For changing their medication + and changing it back when the drug is available again for additional laboratory control for setting the blood pressure etc	5
**Searching for delivery choices from abroad and Clarifying the assumption of costs**	34
Additional contact between the medical practice and the patient After being sent back from the pharmacy	3
Adjusting/convert the dosage	3
Managing the medicine cabinet: Rearrangement and order of medication	3

## Discussion

### Temporal and regional classification of supply shortages

#### Listed international drug supply shortages before the start of the survey period

Table 3 shows the diversity of drug shortages in the last years before the survey period. It can be seen that years ago, not only outside Europe, but already within Europe (Pauwels et al. 2014), drug shortages were registered, especially for anti-infective drugs. This group of drugs was particularly considered in the survey. Another frequently mentioned group are drugs for the treatment of the nervous system.

**Table 3:** International supply shortage before November 2022. List of affected countries, the period of the supply shortage and the situation of the drug shortage

Affected countries	Period	Supply shortage situation
South America, Belgium, Israel, US	2009–2017	From 2009 to 2013, in Belgium 23% of the shortages were drugs for the treatment of the nervous system and 21% of the drugs reported as missing were missing for the treatment of the cardiovascular system From 2013 to 2015, the largest percentage (21%) of supply shortages that was reported in Israel were also drugs for the treatment of the nervous system In the US, in 2013–2015, the drugs for the treatment of the nervous system were also the greatest percentage of supply shortage In 2017, 21% of the supply shortages which were reported in South America were anti-infectives for systemic use. The second largest group of supply shortages were drug products for the nervous system with 17% (Acosta et al. 2019, p. 9)
France	2012–2018	In France from 2012 to 2018, a maximum of ca. 20% of the drugs that were missing belonged for the use as anti-infectives for systemic use. In 2018, ca. 12% of the missing drugs were antiinfectives for systemic use. In 2018, the causes of shortages were mainly material issues, manufacturing issues, and the pharmaceutical market (Benhabib et al. 2020)
US	2018–2019	In a survey from 2018 to 2019, postgraduates from year 2 and above in the departments of anaesthesiology, medicine, and emergency medicine at the University of Chicago and Mayo Clinic were asked about their experiences with supply shortages. 51% of the postgraduates stated that they had regulated relevant shortages on a daily or weekly basis. Additionally, 77% stated that in a quarter of the cases they had no equivalent alternative available (Hantel et al. 2020)
China	2018–2021	Chinese national and provincial lists of drug shortages were analysed. In the time range from 2018 to 2021 17% of the drug shortages that were missed are for treating the cardiovascular system. The second and third largest proportion of drugs that were missed are for treating the nervous system (13.6%) and the antineoplastic and immunomodulating agents (12.1%) It is also striking that the dosage from “injections” (45.3%) was most often missing. (Song et al. 2023)
Estonia, Finland, Norway, Spain, USA	2020	From January to September 2020, 50% of the supply shortages reported in the U.S. were drugs used to treat the nervous system. In Estonia, Finland, Norway, and Spain, the proportion of drugs for the treatment of the nervous system was only about 25%. The share of missing drugs for systemic anti-infective treatment did not exceed 12.5% in any of the 5 countries. In terms of the lack of dosage forms, the USA also differed from the other four countries. The dosage form that was most lacking in the USA was “injectables”. In Estonia, Finland, Norway, and Spain, “tablets” was the dosage form, that was missed the most. (Ravela et al. 2022) (Pauwels et al. 2014)

#### Listed drug shortages in the time queried in the survey: comparison between Germany, Austria, Switzerland, Canada, and the USA

**Fig. 15:**
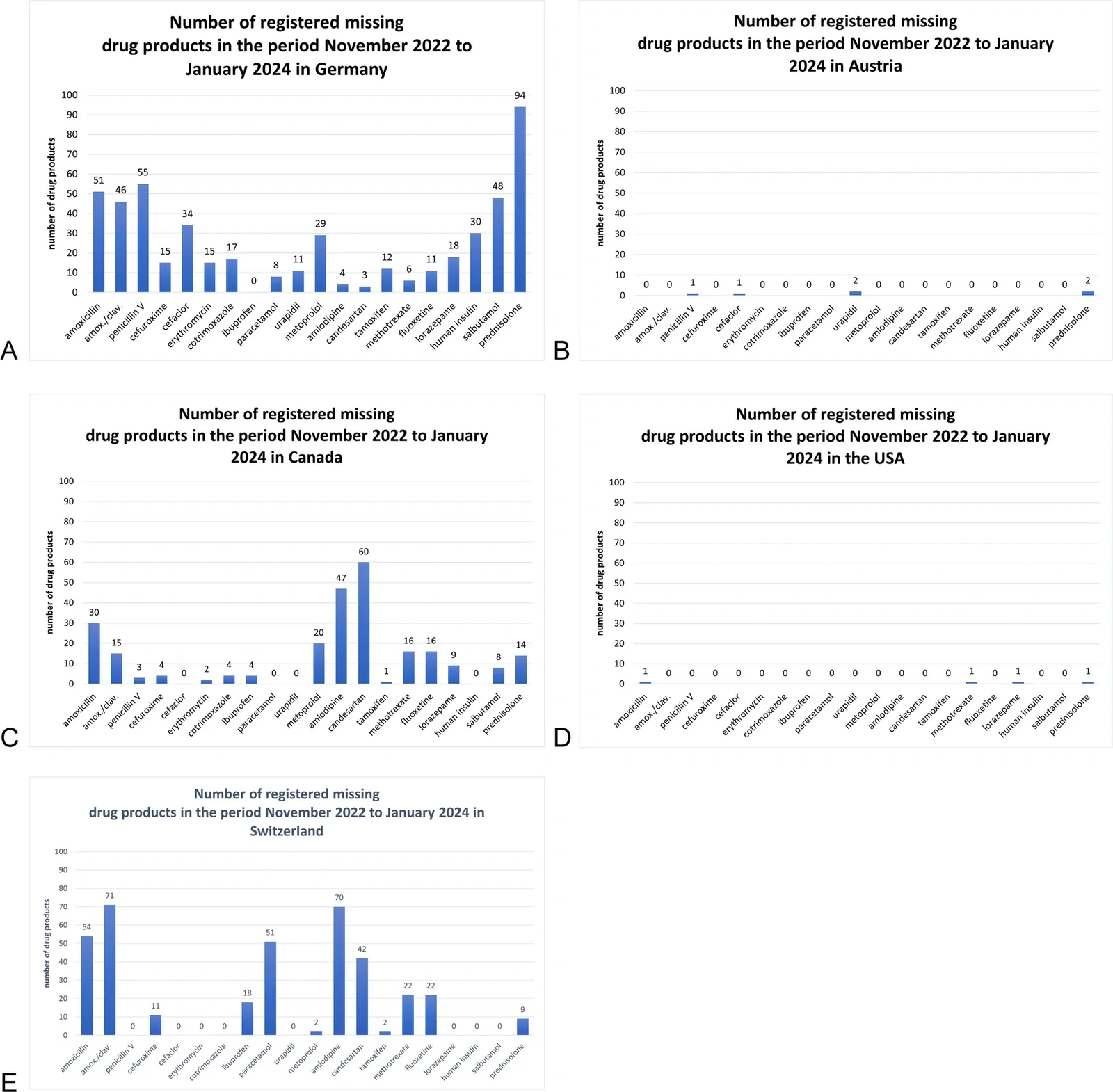
**A**–**E** The number of registered unavailable drug products in the period from November 2022 to January 2024 in Germany (**A**) (Bundesinstitut für Arzneimittel und Medizinprodukte (BfArM) 2025b), Austria (**B**) (Bundesamt für Sicherheit im Gesundheitswesen 2025), Canada (**C**) (Health Canada n.d.), USA (**D**) (U.S. Food and Drug Administration 2025a), and Switzerland (**E**) (Martinelli Consulting GmbH 2025) is shown. The number of missing drug products for the 20 drugs investigated in the survey are described

The time for the comparison is the same as the time queried in the survey. However, the reports of missing drug shortages are not necessarily complete, because not all drug supply shortages have to be reported by the companies. The data for the various countries are summarised in Fig. 15 and Table 4.

**Table 4:** Number of registered unavailable drug product in the databases of the individual countries. Reporting obligation in the individual countries: Germany: Notification in accordance with the resolution of the Pharma Dialogue 2016 on the self-commitment. Austria: Notification on the basis of § 57a Abs. 2 des Arzneimittelgesetzes, BGBl. Nr. 185/1983, last amended by the Bundesgesetz BGBl. I Nr. 104/2019, and the Bundesministeriengesetz-Novelle 2020, BGBl. I Nr. 8/2020. Any restriction of the marketability of a prescription medicinal product in Austria must be reported immediately. A restriction of the ability to sell is considered to be an unavailability of a prescription medicinal product that is expected to exceed two weeks or an insufficient availability of a prescription medicinal product to cover the needs of patients in Austria that is expected to exceed four weeks (Rechtsinformationssystem des Bundes (RIS)  2025). Canada: Since March 2017, manufacturers have been mandated to report actual and expected drug shortages on the Drug Shortages Canada website (Government of Canada 2025). USA: Sect. 506 C of the Federal Food, Drug and Cosmetic Act states that manufacturers must report short-term or permanent production interruptions of prescription drugs to the FDA if they result in a significant disruption to the supply of drugs in the United States (U.S. Food and Drug Administration 2025b). Switzerland: Private initiative to collect information on drug shortages, as the Bundesamt für wirtschaftliche Landesversorgung (BWL/Federal Office for National Economic Supply) only examines those shortages that are relevant for the supply of the country. The current status of the lists of the BWL is nevertheless incorporated into the database of Drugshortage.ch (Martinelli Consulting GmbH 2025)

Country	Number of registered unavailable drug products	Website of the database
Germany	Total number of entries: 4564 Number of entries from Nov. 2022–Jan. 2024: 2791	https://anwendungen.pharmnet-bund.de/lieferengpassmeldungen/faces/public/meldungen.xhtml
Austria	Total number of entries: 580 Number of entries from Nov. 2022–Jan. 2024: 102	https://medicineshortage.basg.gv.at/vertriebseinschraenkungen/faces/adf.task-flow?_document=WEB-INF%2Fmain-btf.xml&_id=main-btf
Canada	Total number of entries: 25,049 Number of entries from Nov. 2022–Jan. 2024: 4208	https://www.drugshortagescanada.ca/
USA	Total number of entries: 107 Number of entries from Nov. 2022–Jan. 2024:/	https://dps.fda.gov/drugshortages
Switzerland	Total number of entries:/ Number of entries from Nov. 2022–Jan. 2024:/ Current open supply shortages: 755	https://www.drugshortage.ch/index.php/suche-nach-abgeschlossenen-lieferengpaessen/

##### Comparing the listed drug supply shortages with the perceived drug supply shortages assessed by the survey participants

Two thousand seven hundred ninety-one missing drug products were registered form November 2022 to January 2024 in Germany. Of the 20 drugs studied here, most products concerned prednisolone. Many products with antibacterial drugs were also reported. Products containing amoxicillin, amoxicillin + clavulanic acid, and penicillin V were reported the most. Compared to the extent to which medical doctors were affected (Fig. 6), a similar picture emerges.

Although 94 prednisolone products were on the shortage list (Fig. 15A), less than 10% of the general practitioners were strongly or very strongly affected by the supply shortage of prednisolone (Fig. 6A). A possible explanation for this lack of effect of drug shortage on daily practice may be that glucocorticoids are generally perceived as “dangerous” and, therefore, prescribed cautiously anyway. For example, in the systematic review “Topical corticosteroids phobia in atopic dermatitis”, the fear of topical steroids is reported which leads to a negative impact on the adherence of the patients additionally (Li et al. 2017). Supply shortages such as that of ibuprofen are not registered. But according to the survey, 59% of paediatricians were strongly or very strongly affected by shortages of ibuprofen (Fig. 6C). Thus, here, we are dealing with two opposite cases of discrepancies between listed drug shortage and perceived drug shortage. The discrepancies between our survey data (Fig. 6) and registrations of drug shortages (Fig. 15) suggest that the registrations are, on the one hand, incomplete and, on the other hand, not necessarily practically relevant.

Therefore, it is interesting to compare the number of registered unavailable drug products with other countries. From the 20 drugs from the survey, only a few numbers of drug products were registered by the Federal Office for Safety in Health Care in Austria (Fig. 15B). This shows a discrepancy between the registered drug shortages and the actual extent to which medical doctors in Austria were affected. For example, more than half of the survey participants (52%) were strongly or very strongly affected by the supply shortage of amoxicillin + clavulanic acid and penicillin V (Fig. 8) but only one drug product with the drug penicillin V was registered to be not available in the period of the survey. In the case of Austria, it suggests an underreporting of drug shortage by the official authorities.

The numbers of registered drug products per drug in Canada differs from the German and Austrian registrations (Fig. 15C). In Canada, many unavailable antihypertensive drug products for candesartan, amlodipine, or metoprolol have been reported. Compared to the German registrations, fewer antibacterial drugs were reported in Canada during the period.

The reports of the products not available in Switzerland also show differences compared to the German and Austrian reports (Fig. 15E). Strikingly, 51 drug products with paracetamol were registered as unavailable. In Germany, on the other hand, only 8 drug products with the drug paracetamol were registered and none in Austria. A similarity can be found for amoxicillin and amoxicillin + clavulanic acid. Fifty-one drug products with the drugs amoxicillin were registered as unavailable in Germany, similar to 54 registered drug products in Switzerland. There are similar tendencies with amoxicillin + clavulanic acid in Germany and in Switzerland with registered unavailable drugs.

For the USA, very few drug products (4) with the 20 drugs from the survey were registered as unavailable in the period from November 2022 to January 2024 (Fig. 15D). The register of the American Society of Health-System Pharmacists (ashp) also contains a similar number of reports for the 20 drugs for the specified period (ashp pharmacists advancing healthcare 2025).

Many possible factors could contribute to this difference in the registration of supply shortages between Germany and the USA. First of all, the reporting systems are only comparable to a limited extent. Furthermore, the demand for the drugs in the respective countries can be variable. The supply chains of the drugs can be different and can have different effects on supply shortages. The differences between the German and American pharmaceutical markets could also cause a difference in drug shortages.

### Classification of the physicians' involvement in drug shortages of the 20 drugs from the survey

#### How medical doctors have been affected by the supply shortage

General practitioners were most affected by the supply shortages of penicillin V amoxicillin and amoxicillin + clavulanic acid. Internal medicine specialists were most affected by the supply shortages of penicillin V, lorazepam, and amoxicillin. More than half of the general practitioners were not strongly or very strongly affected by the supply shortages of the 20 drugs except for penicillin V. The same patterns appear for the physicians working in internal medicine. More than half of the internal physicians were not strongly or very strongly affected by the supply shortages of the 20 drugs without any exceptions. Paediatricians show a different image. For amoxicillin, penicillin V, and ibuprofen more than half of the paediatricians rated to be strongly or very strongly affected by the supply shortages of these drugs.

Looking at the comparison of internal physicians working in practices in comparison to internal medical physicians working in clinics (Fig. 7), one big difference could be seen. Forty percent of the internal physicians working in practices were strongly or very strongly affected by the supply shortage of penicillin V. In comparison to this, only 5% of the internal physicians working in clinics were strongly or very strongly affected by the supply shortage of penicillin V.

German general practitioners were more strongly or very strongly affected by the supply shortages of penicillin V, erythromycin, urapidil, and salbutamol than the Austrian medical doctors. For amoxicillin + clavulanic acid, cefaclor, and prednisolone, the Austrian general practitioners were more affected by the supply shortages than the German physicians.

Additionally, regional differences within Germany could be seen for the supply shortages of salbutamol and urapidil. The percentage of general practitioners being strongly or very strongly affected by the supply shortage of salbutamol was significantly higher for Lower Saxony than for Thuringia. But the percentage of physicians being affected by the supply shortage of urapidil was significantly higher for Thuringia than for Lower Saxony.

#### Increased demand as source for supply shortages: case studies

Pharmaceutical companies cite an increased demand for the respective drug as the cause of the supply shortage. This can be seen in the database of the BfArM (Bundesinstitut für Arzneimittel und Medizinprodukte (BfArM) 2024).

##### Penicillin V and amoxicillin

The antibacterial drugs penicillin V and amoxicillin are often used for treatment of tonsillopharyngitis, acute otitis media, acute sinusitis, or community acquired pneumonia (Abele-Horn et al. 2019, pp. p.28–64). The perceived supply shortage of the drugs (Fig. 6A–C) matches with the rising incidence of acute respiratory diseases (ARE) since November 2022. In the Flu Web Weekly Report of the RKI, this increasing incidence of acute respiratory diseases per 100,000 inhabitants per calendar week is plotted (Robert Koch Institut: Buchholz U, Buda S, Lehfeld AS, Loenenbach A, Prahm K, Preuß U, Haas W, AMELAG-Team 2024, p. p. 3). The supply shortages of penicillin V and amoxicillin among paediatricians can be explained with the data from the Flu Web Weekly Report of the RKI because the incidence of acute respiratory diseases was higher for children in comparison to the adults in the time range of the survey (Robert Koch Institut: Buchholz U, Buda S, Lehfeld AS, Loenenbach A, Prahm K, Preuß U, Haas W, AMELAG-Team 2024, p. p. 3). Although most of the acute respiratory diseases have a viral genesis, the RKI (Robert Koch Institut) already stated in its Weekly Situation Report on the Coronavirus Disease Robert 2024) (COVID-19) on 22.12.2022 that the number of bacterial infections increased: “The current very strong circulation of respiratory viruses is accompanied by increased bacterial infections of the respiratory tract. The number of group A streptococcal infections and pneumococcal infections is expected to continue to increase. This poses an additional challenge for the healthcare system” (RKI 2022, p. p.5). Encouraging more conservative and rational prescription of penicillin V and amoxicillin may help mitigate perceived drug shortages. In fact, in recent studies, we have provided compelling evidence for irrational antibacterial drug prescribing practises in Germany. For example, a strong association was found between falling drug prices and rising prescriptions (Bindel and Seifert 2024). However, further studies are needed to show a direct connection between irrational antibacterial drug prescribing practises and resulting supply shortages.

##### Lorazepam

**Fig. 16:**
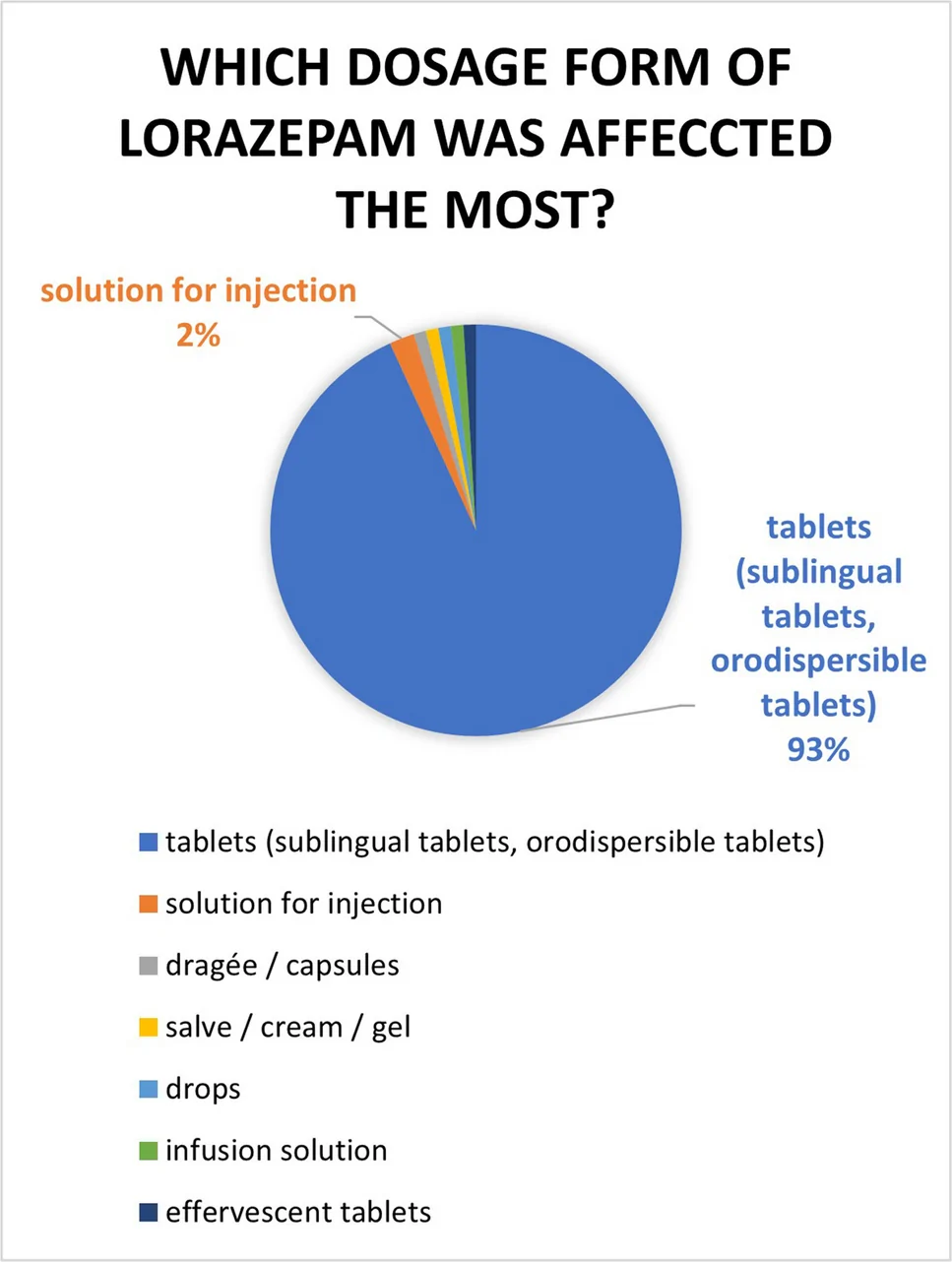
Distribution of the dosage forms of lorazepam that were missing

General practitioners were also more affected by the supply shortage of lorazepam than from other drugs. The dosage form that was missed the most were tablets (Fig. 16).

Lorazepam belongs to the group of benzodiazepines, which are used for restlessness, anxiety, and tension as well as psychosomatic complaints. (Geisslinger et al. 2020, p. p. 229). In a long-term observation of the mental health of the adult population in Germany by the RKI, an increase in the proportion of the adult population with stress from anxiety symptoms can be seen from April 2021 with 9.7% to 14.8% in March 2024.(Robert Koch Institut 2024). In future studies, it would be interesting to analyse if the rising incidence of anxiety symptoms since 2022 could contribute to the supply shortage of lorazepam. Lorazepam is not suitable for long-term of anxiety problems anyway because of the risk of tolerance, dependence, and adverse drug reactions (Michelini et al. 1996).

##### Urapidil

**Fig. 17:**
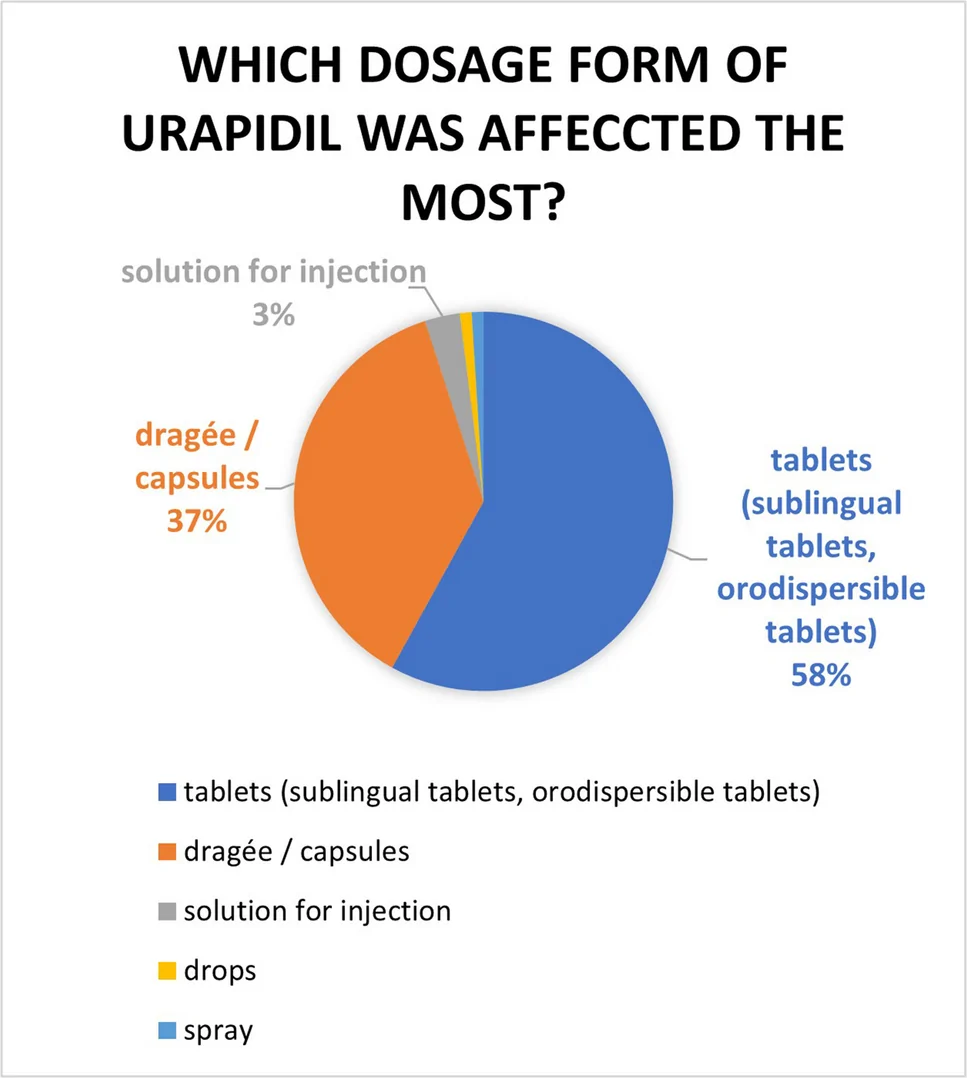
Distribution of the dosage forms of urapidil that were missing

General practitioners were affected to varying degrees by supply shortages of antihypertensive drug, urapidil being prominent (Fig. 6A). Fifty-eight percent of the general practitioners working in Thuringia stated to be affected by the supply shortage of urapidil whereas the percentage in Lower Saxony was much lower with 16%. The dosage form that was missed the most was the tablet form with 58% and capsules with 37% (Fig. 17).

In the guidelines for antihypertensive therapy, urapidil is not recommended as long-term medication (AWMF online 2023, p. p. 26). Urapidil is only found as an emergency medication for intravenous application (AWMF online 2023, p. p. 33). Urapidil is also recommended in the therapy of the very rare pheochromocytoma or as second-line therapy for benign prostate hyperplasia. Because the dosage forms that were missed the most are for oral application and not for intravenous application based on the guidelines, physicians cannot have missed urapidil as therapy for hypertonia.

**Fig. 18:**
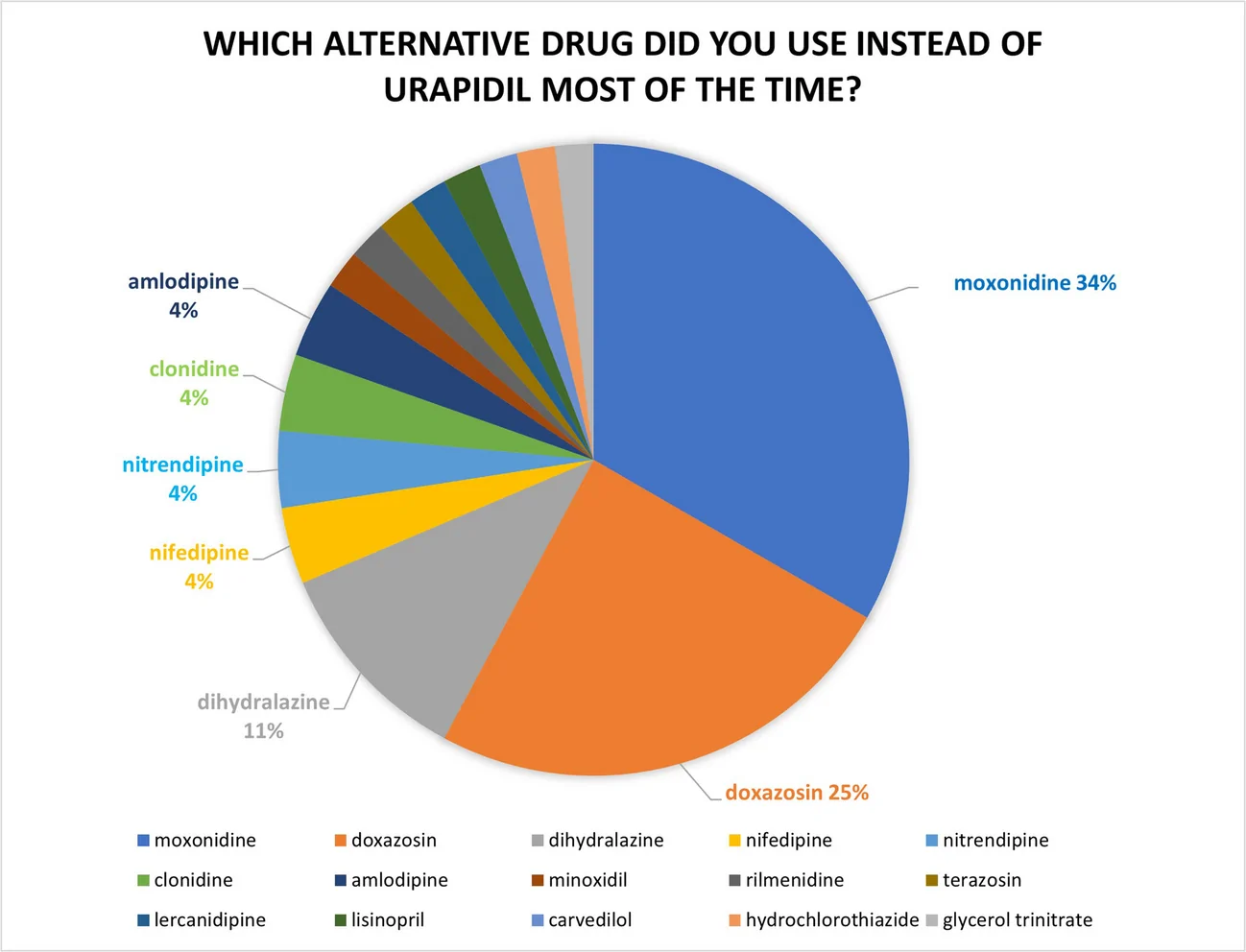
Distribution of the alternative drugs that were used instead of urapidil

The alternative drugs physicians used instead of urapidil are shown in Fig. 18. The most frequently chosen drugs were moxonidine (34%), doxazosin (25%), and dihydralazine (11%). Of these 3 drugs, doxazosin, with a recommendation level of 0 (open recommendation), is found in the guideline for benign prostate syndrome. Moxonidine and dihydralazine are not mentioned (Deutsche Gesellschaft für Urologie e.V. 2023). Thus, it appears that urapidil was mostly used for long-term therapy for hypertension although it is not mentioned for this purpose in the guidelines. Accordingly, in the case of urapidil, we seem to have a case of pseudo supply shortage due to inappropriate prescription practice. We have no explanation why urapidil is prescribed for long-term therapy of hypertension.

### Overview of the additional time expenditure

#### Summary of the time expenditure

Three sources of additional time expenditure were queried: phone calls to the pharmacy, searching for alternatives and speaking with the affected patient. Within the specialty the physicians rated similar amounts of additional time for the three reasons, on average. But the additional time that was needed varies between the specialties. From the general practitioners and internal medicine physicians the largest percentage needed 5 min additional time per drug for each of the three reasons (calls with pharmacy, searching for alternatives, talking with the patient). The most additional time burden per affected drug was recognised in haematology/oncology with 46 min, followed by psychiatry (37 min), nephrology (37 min), and general medicine (28 min). The two most commonly mentioned additional sources for additional time expenditure are changing prescriptions and communicating with other instances.

### Time expenditure for physicians in comparison to pharmacists

Drug shortages have an impact on the well-being of pharmacists in a public setting which is also amplified by the lack of personnel (Beuriot and Crunenberg 2024).

On 8 October 2024, the Pharmacy Climate Index 2024 was presented at a press conference for the German Pharmacists’ Day. Survey results presented in this context also address the topic of supply shortages and the associated additional effort. More than half of the pharmacists (63%) rated the additional effort here as high, very high, or extremely high (Bundesvereinigung Deutscher Apothekerverbände (ABDA) 2024, p. p. 43). Thus, both physicians and pharmacists needed a lot of additional time due to supply shortages. For pharmacists, “the biggest challenges in managing supply shortages are patient communication (80.4%), consultation with physicians (76.0%), and availability requests from wholesalers (75.8%)” (Bundesvereinigung Deutscher Apothekerverbände (ABDA) 2024, p. p. 42). Thus, both physicians and pharmacists both needed a lot of time talking with each other and with patients.

## Limitations

First, a response bias arises from the fact that most participants that answered the survey were from the field of general medicine. Therefore, data without a specialty filter cannot be used to draw conclusions about the general medical profession. The regional imbalance because of the lack of the willingness to distribute the survey might lead also to a response bias. However, in general, it can be assumed that country effects are dominating regional effects. Significant regional effects that have been found during analysis were shown in chapter 2.4. But of course, the small coverage of several regions could leave other regional effects hidden. Second, it can be assumed that physicians who were severely affected by supply shortages probably tended to participate in this survey rather than those who were not affected by the mentioned supply shortages. Third, the survey asks participants questions about events from the past. This can lead to a recall bias and, if necessary, a reporting bias. Fourth, not all participants completed the questionnaire. This creates a loss to follow-up bias. Fifth, the unequal distribution of participants per state could also influence the survey results.

## Conclusions

The survey shows the serious consequences of the supply shortages for the medical profession. The impact of shortages among the 20 drugs assessed was very different, both regionally within Germany and Austria, and among various medical specialities. Penicillin V and amoxicillin emerged as the most critical drugs.

Supply shortages lead to substantial time burden for doctors. A different time burden depending on the discipline was also noticed, haematology/oncology, psychiatry, and nephrology being particularly vulnerable. Searching for alternatives, calling the pharmacy, and conversing with the patient all contribute to the time burden. In order to minimise the additional time required to search for alternatives, the publication of lists of recommended alternative drugs by expert groups of the respective specialties could be useful. For example, a recommendation on alternative calculated oral antibiotic therapies in outpatient paediatrics in the event of supply shortages of penicillin, amoxicillin or amoxicillin beta-lactamase inhibitors in cooperation with the Professional Association of Paediatricians and Adolescents (bvkj), the German Society for Paediatrics and Adolescent Medicine (DGKJ), and the German Society for Paediatric Infectiology e.V.. was released (Berufsverband der Kinder- und Jugendärzte e.V. (bvkj.) et al. 2025). Such recommendations could make it easier for physicians to find the right alternative in the event of a shortage and minimise the additional time required. Online drug product availability check integrated in the medical practice management software and hospital information systems could maybe also reduce the extra effort especially for calling the pharmacy and changing prescriptions.

We observed discrepancies between officially reported drug shortages and drug shortages reported by survey participants. This discrepancy may suggest underreporting in official registries, or that physicians experience availability issues that are not captured by formal records. Thus, it will be important to improve the registration systems for drug shortages. Ultimately, reporting data bases should accurately reflect the practically relevant drug shortage. In this way, important stakeholders involved, e.g. pharmaceutical companies, health insurances, doctors, pharmacists, learned societies issuing guidelines and the public media can respond more diligently and rationally to drug shortages.

The identification of particularly critical drugs, vulnerable medical specialities, and specific time-consuming factors offer starting points for improving the consequences of drug supply shortages. Forecasting future drug consumption (Pall et al. 2023), specific pharmacological education for all medical specialities on drug alternatives, patient education on the basic principles of pharmacology, and training of physicians and pharmacists in communication techniques (Mashal et al. 2024) are all important elements to solve the consequences resulting from drug supply shortages. In response to the drug shortages in Germany, we have recently begun to systematically project future consumptions of antibacterial drugs using sophisticated mathematical models (Bindel and Seifert 2025). Lastly, rational drug prescribing is important for avoiding drug shortages due to “increased demand”.

Finally, as a response to drug supply shortages, the Deutsche Gesellschaft für Innere Medizin (DGIM, German Society for Internal Medicine) has recently defined a list of essential drugs (DGIM Deutsche Gesellschaft für Innere Medizin 2025). It is hoped that other medical societies follow this role model. With a consensus on essential drugs, procurement of sufficient quantities of these drugs will become more transparent and easier for all stake holders, e.g. drug companies, federal agencies, hospitals, practitioners, health insurances and pharmacies.

## Supplementary Information

Below is the link to the electronic supplementary material.

ESM 1 (DOCX 24.1 K)

## Data Availability

All source data of this study are available from the authors upon reasonable request.

## Abbreviations

RKI: Robert Koch Institute
BfArM: Bundesinstitut für Arzneimittel und Medizinprodukte (Federal Institute for Drugs and Medical Devices)
MHH: Medizinische Hochschule Hannover (Hannover Medical School)
BASG: Bundesamt für Sicherheit im Gesundheitswesen (Österreich) (Federal Office for Safety in Health Care (Austria))
