# Supplementary material for: Drug supply shortages and their consequences for the medical profession: a survey in Germany and Austria
Source: Naunyn Schmiedebergs Arch Pharmacol. 2026 Jun 6;399(11):18157–79. doi: 10.1007/s00210-026-05484-6 (PMC13503409; doi:10.1007/s00210-026-05484-6)
Supplement: Supplementary file 1 [file 210_2026_5484_MOESM1_ESM.docx]

**Supplemental data**

**Drug supply shortages and their consequences for the medical profession: A survey in Germany and Austria**

**Julia Maria Rotter and Roland Seifert**

| German survey distributors | Austrian survey distributors |
| --- | --- |
| Ärztekammer (German medical chamber)   - Thüringen (Thuringia) - Saarland - Westfalen-Lippe - Bremen (Hanseatic City) - Schleswig-Holstein - Mecklenburg-Vorpommern (Mecklenburg-Western Pomerania) | Österreichische Ärztekammer  (Austrian medical chamber)   - Burgenland - Oberösterreich (Upper Austria) - Salzburg |
| Kassenärztliche Vereinigung (association of Statutory Health Insurance Physicians)   - Hamburg (Hanseatic City) - Schleswig- Holstein - Mecklenburg-Vorpommern  (Mecklenburg-Western Pomerania) - Niedersachsen (Lower Saxony) - Sachsen (Saxony (Free State)) | Vereinigung österreichischer Ärzte  (Association of Austrian medical doctors) |
| Hausärzteverband Westfalen-Lippe  Hausärzteverband Saarland  Hausärzteverband Niedersachsen | - Österreichisches Institut für Allgemeinmedizin Kärnten (öifam) (Austrian institute for general medicine Carinthia) - Tiroler Gesellschaft für Allgemeinmedizin (TGAM) (Tyrolean Society for General Medicine) - Oberösterreichische Gesellschaft für Allgemeinmedizin (Obgam) (Upper Austrian Society for General Medicine) |
|  | Der Arzneimittelbrief (the medication letter) |
| Deutsche Gesellschaft für Innere Medizin  (German society for internal medicine) |  |
| Berufsverband Deutscher Internistinnen und Internisten  (Professional association of German internists) |  |
| Institut für Allgemeinmedizin und Palliativmedizin der MHH  (Institute for general and palliative medicine at the MHH |  |

**Table S1**: List of German and Austrian survey distributors

| Question number | Question | Answers |
| --- | --- | --- |
| Question block 1 General questions | | |
| 1.1 | Enter the state of your place of work | Drop-Down menu  with federal states of Germany and Austria |
| 1.2 | Select your field of study | Drop-Down menu  with specializations |
| 1.3 | In which setting do you work? | practice  clinic  company  other workplace |
| Question block 2  Individual evaluation of all 20 drugs | | |
| 2.1 | Please assess the extent to which you have been affected by supply shortage for the following drugs | Rating each of the 20 drugs with a number from 0 to 5  1=not affected  2=slightly affected  3=moderately affected  4=strongly affected  5=very strongly affected  I can't judge |
| Question block 3  Enquiries about valuation in 2.1  All drugs present in 2) **x >= 3** are supplemented with the following questions per drug  "For the drug *XYZ,* you have selected 3 or higher; please answer the following questions only in relation to the drug ***XYZ***" | | |
| 3.1 | 3.1) Which dosage form of drug XYZ was most affected? | Choosing a dosage form |
| 3.2 | How did you deal with the supply shortage of the drug ***XYZ*** with the dosage form selected in 3.1 for the largest number of your patients? | - I have prescribed a different dosage form of the drug **XYZ** ." - I have prescribed a different dosage of the drug XYZ - I discontinued the drug XYZ earlier as planned without an alternative - I did not prescribe the drug from the beginning and waited - I have prescribed a different drug |
| 3.3 | Which alternative drug(s) have you chosen? | Free text field |
| 3.4 | Evaluate the treatment success of the alternative compared to the originally intended drug. | 0-5 points  0=very bad  5=very good |
| Question block 4  Time spent due to supply shortages | | |
| 4.1 | Please indicate how much additional time you had for the respective tasks per drug concerned  Tasks:  Telephone calls with the pharmacy  Search for alternatives for the Pat.  Conversation/Enlightenment of the Pat. | Evaluation of the 3 tasks with one of the time indications:  Approx. 0 min per drug  Approx. 5 min per drug  Approx. 10 min per drug  Approx. 15 min per drug  Approx. >=20 min per drug |
| 4.2 | Did you have any additional time expenditure due to something else that has not yet been mentioned above? | Free text field to give another reason for additional time expenditure |

**Table S2**: Questionnaire of the survey divided into 4 question blocks
